# Transforming Microbial Genotyping: A Robotic Pipeline for Genotyping Bacterial Strains

**DOI:** 10.1371/journal.pone.0048022

**Published:** 2012-10-29

**Authors:** Brian O’Farrell, Jana K. Haase, Vimalkumar Velayudhan, Ronan A. Murphy, Mark Achtman

**Affiliations:** Environmental Research Institute, University College Cork, Cork, Ireland; The Roslin Institute, University of Edinburgh, United Kingdom

## Abstract

Microbial genotyping increasingly deals with large numbers of samples, and data are commonly evaluated by unstructured approaches, such as spread-sheets. The efficiency, reliability and throughput of genotyping would benefit from the automation of manual manipulations within the context of sophisticated data storage. We developed a medium- throughput genotyping pipeline for MultiLocus Sequence Typing (MLST) of bacterial pathogens. This pipeline was implemented through a combination of four automated liquid handling systems, a Laboratory Information Management System (LIMS) consisting of a variety of dedicated commercial operating systems and programs, including a Sample Management System, plus numerous Python scripts. All tubes and microwell racks were bar-coded and their locations and status were recorded in the LIMS. We also created a hierarchical set of items that could be used to represent bacterial species, their products and experiments. The LIMS allowed reliable, semi-automated, traceable bacterial genotyping from initial single colony isolation and sub-cultivation through DNA extraction and normalization to PCRs, sequencing and MLST sequence trace evaluation. We also describe robotic sequencing to facilitate cherrypicking of sequence dropouts. This pipeline is user-friendly, with a throughput of 96 strains within 10 working days at a total cost of < €25 per strain. Since developing this pipeline, >200,000 items were processed by two to three people. Our sophisticated automated pipeline can be implemented by a small microbiology group without extensive external support, and provides a general framework for semi-automated bacterial genotyping of large numbers of samples at low cost.

## Introduction

Industrial laboratories and institutional core facilities increasingly employ robotics supported by sophisticated bioinformatics for fluidic manipulations [1]. Automation of laborious, repetitive and time-consuming processes is making rapid progress in areas such as food production [2]–[4], pharmaceutical screening of biologically active compounds [5], [6] and genomic sequencing [7]. The possibilities for automation and data management are illustrated by the “Robot Scientist”, which formulates hypotheses, designs experiments and then executes them, and analyzes and stores the results without human intervention [8], [9]. Such developments emphasize a growing interest in robotic systems as experimentation tools where user-friendly plasticity is at least as important as the degree of throughput.

While the initial manual steps in diagnostic microbiology can now be performed by commercial automated systems [10], the implementation of fluidic robotics and its integration into a laboratory information management system (LIMS) remains exotic in standard microbiology. Most smaller microbiological laboratories rely heavily on traditional, manual techniques, and often lack a multi-user, electronic data management system [11]. Experiments, sample locations and data are usually documented in project-specific lab books or in unstructured and uncoordinated spreadsheets. Such practises can result in inefficient communication; loss of data; and issues with knowledge transfer, particularly in research groups with rotating staff and temporary visitors. Data security can be strengthened by the use of structured, multi-user databases for recording sample locations and experimental details within the context of a central LIMS. The use of a LIMS is critical when large amounts of data are processed, and also supports data mining.

Multiple open source LIMS have been recently described which support proteomics [12], high throughput screening of small molecules and RNAi [13], managing a DNA sequencing facility [14], fungal taxonomy via DNA barcoding [15], microsatellite genotyping of crop plants [16] and management of genetically manipulated mouse colonies [17]. However, we are not aware of a LIMS which can be readily integrated by microbiologists, especially by groups lacking a dedicated IT specialist.

In 2007, we used the opportunity of starting a new research laboratory with a modestly sized team of biologists to develop a semi-automated, medium throughput genotyping pipeline embedded within a system for the reliable tracking of samples and their locations. Here, we describe this pipeline in sufficient detail that it could be partially implemented, or fully reproduced and expanded, by a microbiology or molecular biology group with only limited IT support, as well as a brief video showing its main features (http://youtu.be/R3FgGVZi3Ik).

## Materials and Methods

### Software

ItemTracker 3.1.49 (ItemTracker, South Nutfield, UK) was used to store information on items and their locations with the modifications described here (File S1). The back-end of ItemTracker consisted of a multi-user Microsoft SQL database. Sequence analysis and storage of detailed strain information was performed in Bionumerics 6.5 (Applied Maths, Sint-Martens-Latem, Belgium) linked to a PostgreSQL 8.1.11 back-end. The CherryPicking database consists of one SQL table in PostgreSQL (Table S1). The LIMS consisted of these three sets of databases, glued together by scripts in Python or the Bionumerics scripting language (Table S2). In particular, Python Scripts A1 and B4 provide general functions to interact with ItemTracker, and are used by other scripts for updating ItemTracker, or retrieving data from it. However, all databases are directly accessible *via* Python scripts using the open source pyodbc module (http://code.google.com/p/pyodbc/). Notched box and whiskers and the scatter plot were calculated with R version 2.10.1 [18].

### Computer Hardware

Multi-user access to ItemTracker and BioNumerics is provided by clients on Windows-based PCs. The database servers were located on a small Unix cluster within the same sub-network with SAN HDDs and daily incremental tape backup.

### Liquid Handler Systems

Combinations of fluidic robots and other instruments that enable individual stages within our pipeline are referred to here as ‘liquid handler systems’ (LHS). LHS1 consists of a 96 tip Apricot pipettor (Xpp-96H, FluidX, Cheshire, UK), a 96 tube capper/decapper (Xsd-96(F), FluidX), a SCARA robot arm (PAA, Farnborough, UK), a 1-D bar-code reader (MS7120, Orbit, Honeywell International, Morristown, New York, USA) and a 96 tube 2-D bar-code reader (Xtr-96 MKII, FluidX) (Fig. S1a). LHS1 is built into a class 2 safety cabinet (CAS-CL2, Contained Air Solutions, Manchester, UK) to ensure sterility. LHS1 was used for dispensing sterile media into 1.4 ml 2-D bar-coded tubes (65-52330-Z6-LS, FluidX) and for sub-culturing bacteria from racks of 2-D tubes into other racks and deep well plates (DWP) (60–2332, FluidX). DNA extraction was performed with LHS2 (X-tractor Gene CAS-1820, Corbett [now QIAxtractor, 9001794, Qiagen]) (Fig. S1b). LHS3 (Multidrop Combi, Thermo Scientific), a peristaltic pump based system, was used for rapid and accurate dispensing of 2–110 µl volumes into 96- or 384-well plates (Fig. S1c). LHS4 comprises a cabinet which is secured during use, containing a pipetting robot (AJV001, Janus, PerkinElmer) with two arms, one with an eight-tip head to aspirate and dispense volumes into 96- and/or 384-well plates, and the second for gripping and moving labware. The deck of this robot contains space for plates and reservoirs, a wash station, cooling devices (7100046 Thermoshake, Inheco plus 8900019 TEC Control Shaker, Inheco) for standard 2 ml micro-centrifuge tubes (2320-00, Anachem) and a 1-D bar-code reader (MicroScan) (Fig. S1d). The cabinet also includes a fluorimeter (Victor plate reader, 1420-041, Perkin-Elmer), a 96 tube 2-D bar-code reader (Xtr-96 MKII, 20-2120, FluidX), and a Luminex 200 analyzer (LX200-XPON, Luminex), which are all located off-deck but can be accessed by the gripper arm. Elsewhere in the same room is a 96 tube capper/decapper (Xsd-96 Pro, FluidX) which is manually operated to cap and decap 2-D bar-coded 0.5 ml tubes (65-52325-L-S, 65-53111-S, FluidX) which are used for storing DNAs. LHS4 was used for DNA normalization, PCR and sequencing reaction setup.

### Cultivation and Storage (Module A)

Bar-coded labels (THT-59-492-10, Brady) were printed with a Brady label printer (BP1344) using Script A7. These were affixed to agar plates (tryptone soya agar, W11245, Fannin, Ireland for *Salmonella enterica* and brain heart agar, CM375B, Oxoid for *Listeria*) on which bacteria were streaked to single colonies, and to the original containers in which the bacteria had been shipped. The agar plates were incubated at 37°C. After one day (*S. enterica*) or one to seven days (*Listeria*), single colonies were manually inoculated into 2-D bar-coded 1.4 ml screw-capped tubes (2D tubes) filled with 750 µl of broth (*S. enterica*: Oxoid tryptone soya broth [TSB], CM0129; *Listeria*: Oxoid brain heart infusion broth [BHI], CM225B). (Some experiments were performed with other volumes ranging from 500–1000 µl). These liquid media were supplemented with 36 mM K_2_HPO_4_, 13.2 mM KH_2_PO_4_, 0.4 mM MgSO_4_, 1.7 mM C_6_H_5_Na_3_O_7_·2H_2_O, 6.8 mM (NH_4_)_2_SO_4_ and 4.4% (v/v) glycerol, which does not inhibit growth but allows subsequent freezing of cultures without any further manipulations. Prior to manual inoculation, the 1.4 ml 2D tubes were filled with liquid media using LHS1 (File S2) and incubated overnight to ensure sterility. An electrical screwdriver (Xmsd-1, FluidX) and bar-code reader (Xtr-1, FluidX) were used for manual capping/decapping and to read bar-codes from single 2D tubes, respectively. The inoculated tubes were assembled in bar-coded 96-well tube racks and the racks were incubated with shaking in a Titramex 1000 incubator (AGB Scientific) until turbid growth, at which point they were stored at −80°C in metal stands (Xrt-1058, FluidX). Control experiments showed that after such cultivation, bacterial survival in these tubes was adequate for sub-cultivation even after 10 sequential cycles of freezing and thawing over a period of 90 days. During the revival of *Listeria* strains from a historical culture collection [19], we independently confirmed the genus assignment by growth on selective agar (CM856, SR140E, Oxoid) and stored these results in ItemTracker (Table S2 Scripts A12–A13). The workflow for reviving these *Listeria* strains, e.g. choosing alternative original cultures in cases of contamination or no growth, was streamlined by Scripts A14–A15.

### Automated Sub-cultivation, DNA Extraction and Normalization (Module B)

Prior to sub-cultivation with LHS1 from frozen 96-well tube racks, they were defrosted on a thawing station (Xtt-96, FluidX). For *S. enterica*, 50 µl of thawed culture was used to inoculate 700 µl of previously dispensed sterile liquid TSB plus freezing supplements in 1.4 ml 2D tubes, and 25 µl aliquots were used to inoculate wells in a DWP each containing 425 µl of TSB without additives (File S2, Script B1). For *Listeria,* 50 µl culture were used to inoculate 500 µl of previously dispensed BHI plus freezing supplement in 2D tubes and 700 µl of BHI per well of a DWP. Other combinations of volumes were also used on occasion (File S2) but the total inoculum removed from the parent tubes needs to be large enough (>50 µl) to account for potential slight differences of liquid heights between tubes because LHS1 is not capable of liquid-level sensing. Tips were automatically decontaminated by LHS1 with disinfectant (Virkon, 330003, DuPont, US) after the procedure. Inoculated DWPs were manually sealed under aseptic conditions with semi-permeable seals (41-1013, FluidX). Both tubes and DWPs were incubated with shaking at 37°C until turbid growth. The sub-cultured tube rack was frozen at −80°C. The DWP was centrifuged at 3,220×g for 5 min (*S*. *enterica*) or 30 min (*Listeria*) at room temperature, and the supernatant was removed by manual pipetting (*S. enterica*) or decanting (*Listeria*). Cell pellets were lysed with 200 µl of CLB buffer (600–500, Genomed, Germany) supplemented with 1 mg/ml Proteinase K (Genomed), and were incubated for 1 hour at 37°C (*S*. *enterica*) or 56°C (*Listeria*). (Note: These minor species-specific procedural differences reflect those in the original manual procedures and were not subsequently optimised.) The DWP containing the lysed cell pellets was placed into LHS2 for automated DNA extraction (Script B17). LHS2 added 400 µl of BQ1 buffer (740923, Macherey-Nagel, Düren, Germany) to the cell pellet using 200 µl filter tips (EVF-180-R-S, Axygen, UK) and the filter plate (7700-2803, Unifilter 800, GE Healthcare) was then pre-wet with 30 µl of BQ1 buffer and incubated for 30 seconds. The lysed cells were mixed ten times by pipetting and 600 µl of cell suspension was transferred onto the filter plate and subjected to vacuum filtration at 25 kPa for 5 min. The filter was washed with 600 µl BW buffer (740922, Macherey-Nagel) per well, and vacuum filtered again for 5 min at 25 kPa. It was then washed twice with 760 µl B5 buffer (740921, Macherey-Nagel) using vacuum filtration for 90 sec at 25 kPa. The sample was dried by applying high vacuum (60 kPa) for 5 min. The purified DNAs were then eluted by adding 150 µl TE buffer (T9285, Sigma-Aldrich) to the filter followed by a 5-min incubation and a 1-min vacuum filtration at 30 kPa. In this case, the eluted DNA was captured in 2-D bar-coded 0.5 ml tubes, which were capped and stored at −40°C.

The concentrations of extracted DNAs were measured with LHS4 and normalized to 3.3 ng/µl; other target concentrations can be defined by the user. 25 μl of extracted DNA was diluted with 50 μl of TE into a new rack of 0.5 ml 2D tubes, mixed and 10 μl was further diluted in 110 μl TE in a 96-well plate. 10 μl of this dilution was mixed with 90 μl of a 1∶450 freshly prepared dilution of PicoGreen (Quant-iT PicoGreen ds DNA assay kit, P7589, Invitrogen). It was important to dilute and dispense the PicoGreen solution immediately before adding DNA because diluted PicoGreen was found to degrade over 10–20 min at room temperature when not bound to DNA, possibly due to exposure to air. As a precaution, pipetting of DNA into the PicoGreen solution by LHS4 and measurement of fluorescence by the Victor is performed in the dark, but we have not tested whether this is necessary. As part of this procedure, LHS4 prepares a standard curve for concentration *versus* fluorescence based on serial dilutions with known concentrations from 0–1 ng/μl of a DNA standard (25250-010 Lambda DNA, Invitrogen). DNA concentrations of the extracted DNAs were calculated based on this standard curve, and the initial 1∶3 dilution in the new rack of 2D tubes was then adjusted to the normalized, final concentration with TE or DNA.

### PCR and PCR Purification (Module C)

PCR reactions were prepared with LHS4, which dispensed 8 µl of master mix consisting of 2 pmol of each primer, 1× PCR buffer including MgCl_2_ (1.65 mM) (18067-017, Invitrogen) and 0.2 µM dNTPs (10297-018, Invitrogen) into four color-coded 384-well plates (4ti00384-BC, 4ti00384/R-BC, 4ti00384/C-BC, 4ti00384/G-BC, FrameStar-384, 4titude, Surrey, UK). Subsequently, 1.5 µl of each normalized DNA (3.3 ng/µl) was dispensed to 14 wells in the 384-well plates. As a conservative measure to help prevent carry over, the tips were washed in a 1∶1 dilution (ddH_2_0) of non-perfumed, commercial thick bleach (Tesco Stores, UK) when changing between each set of eight DNAs. The use of commercial bleach provided a simple and cheap ∼2.5% sodium hypochlorite solution suitable for our purposes. However, a standardised commercial sodium hypochlorite solution may be preferable in clinical laboratories who may want to implement this aspect of the procedure. The bleach wash supplemented our standard flush/wash steps (ddH_2_0) between dispenses. 4 µl of *Taq* polymerase (0.12 U) in 1× PCR buffer (Invitrogen) was added into each well of the 384-well plates by LHS3. The plates were centrifuged for 1 min at 500×g, sealed (Clear Seal 3730, 4ti-0580, 4titude) with a heat sealer (Xts-384, 42-1002, FluidX) and PCR was performed in a thermocycler (PTC0240G, Bio-Rad DNA Engine Tetrad). (Prior to removing seals, all sealed plates from procedures described below were first centrifuged at 500×g for 1 min.) The same PCR programme was used for all seven gene fragments, but differed with the bacterial species: *S*. *enterica* [95°C 5 min; 35 cycles of 95°C 30 sec, 55°C 30 sec, 72°C 30 sec; 72°C 5 min; 4°C ∞] and *Listeria* [96°C 5 min; 35 cycles of 96°C 30 sec, 50°C 30 sec, 72°C 80 sec; 72°C 2 min; 4°C ∞].

The PCR products were purified by adding 2 µl aliquots of “ExoSAP” (1.76 U of Exonuclease I plus 0.35 U of shrimp alkaline phosphotase [Affymetrix]) with LHS3. The PCR plates were centrifuged, sealed and incubated in a thermocycler for 30 min at 37°C, followed by 15 min at 80°C to denature the enzymes.

### Sequencing Reaction (Module D)

3 µl of the cleaned PCR product was diluted by LHS4 into 19 µl H_2_O which had previously been dispensed by LHS3 into a new set of 384-well plates. The plates were sealed, vortexed for 2 min at 2000 rpm (MixMate, Eppendorf) and centrifuged. 2 µl of the diluted PCR products were dispensed by LHS4 into another new set of 384-well plates as templates for sequencing reactions. The templates were centrifuged and dried on a thermocycler at 80°C for 10 min with the lid open. LHS4 was then used to dispense 4 µl of the appropriate sequencing primers (final concentration: 4 pmol) to each well. A 4 µl mixture of BigDye (1/13.3 of recommended concentration) and sequencing buffer (version 3.1, Applied Biosytems) was dispensed with LHS3. The 384-well plates were heat sealed and centrifuged. Sequencing reactions were performed on the thermocycler [96°C, 2 min; 30 cycles of 96°C for 10 sec, 50°C for 5 sec, 60°C for 2 min; 4°C ∞].

Sequencing products (8 µl) were purified with an ethanol precipitation procedure beginning with dispensing 21 µl of 120 mM sodium acetate in 100% ethanol by LHS3 to each product. The plates were sealed, vortexed for 1 min and centrifuged. They were incubated in the dark at room temperature for 45 min and then centrifuged at 3,220×g at 4°C for 1 hour. The supernatant was decanted onto paper, and the plates were centrifuged upside down at 500×g at 4°C for 1 min to remove any remaining droplets. The pellets were washed twice with 30 µl of ice cold 70% ethanol (dispensed with LHS3), consisting of 10 min centrifugation at 3,220×g followed by decanting and centrifugation upside down. The last upside down centrifugation step was extended to 2 min. The products were shipped to a sequencing facility at the Department of Zoology at Oxford University, UK, where they were sequenced with a 3730×l DNA Analyzer (Applied Biosystems). Traces were assembled and analyzed with Bionumerics.

## Results

### Sample Management System

**Figure 1:**
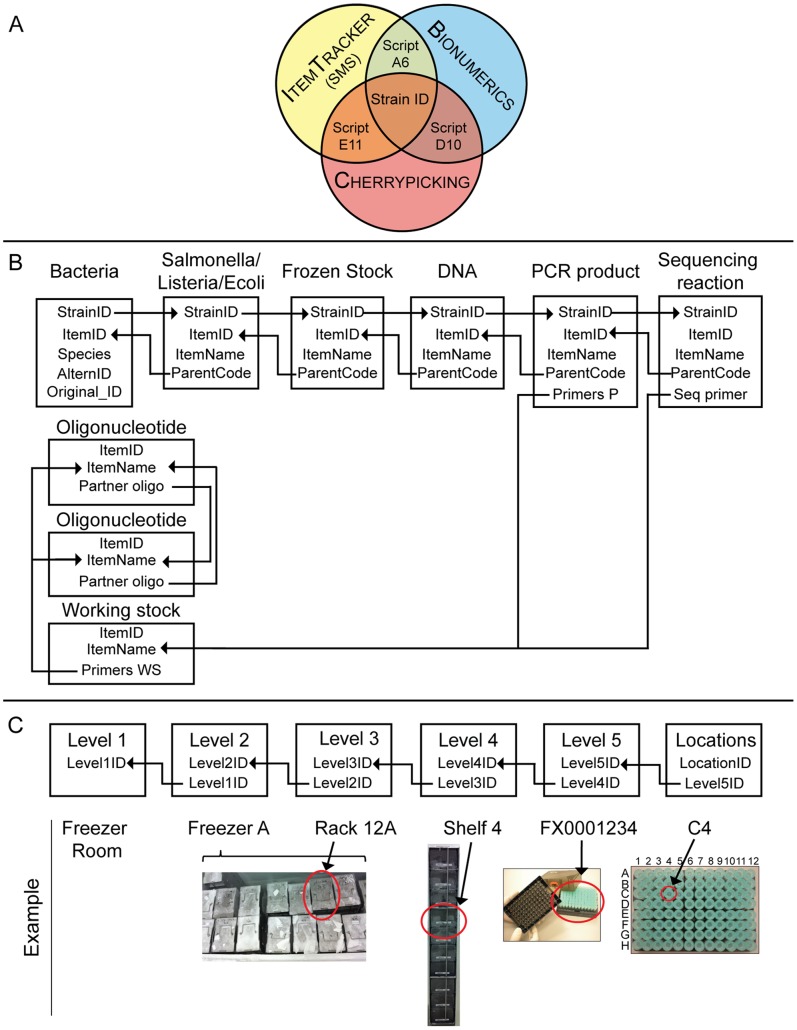
General overview of the LIMS. (A) Overlap between three database systems showing their linking Python scripts and the central item, ‘Strain ID’, which is common to all three. (B) Modified data structure of the ItemTracker SMS, displaying types and hierarchies of data items. Item levels are indicated by rectangles containing important item properties under the Item type designation that was devised for the modified data structure. All item properties are presented in Tables S3 and S4. Arrows indicate hierarchical parent-child relationships, except that ‘StrainID’ is inherited by all decendents of ‘Bacteria’. Each item has a unique ‘ItemID’. Each of the item levels other than ‘Bacteria’ also includes a second, user-understandable, unique designation, ‘ItemName’. (C) Hierarchy of locations in the SMS, including examples of location designations in the lower line.

Over the last four years, we have cultivated and stored numerous bacterial strains, extracted DNAs, and performed multilocus sequence typing (MLST) on the DNAs. In order to organise this information, we used a commercial, generic sample management system (SMS) (ItemTracker) which assigns a unique identifier (ItemID), creation date, user and location to each item and appends a new data entry for each change, thus ensuring tracking of the history of all changes. We extended this SMS for use in a microbiology laboratory by creating a sequential, one-to-many parent-child hierarchy of items, the top of which is a fictitious item ‘Bacteria’, with a unique strain identifier (StrainID) plus alternative designations (Fig. 1b). Each child item possesses a link to its parent, as well as additional level-specific properties (File S1, Table S4), such as a user-friendly identifier, ‘ItemName’.

The next level in the serial hierarchy below ‘Bacteria’ splits into individual species, which were implemented to store species-specific information on the status, the viability and the container type used previously for storage and transport. Status records the progression through stages in the cultivation of bacterial single colonies. Currently, we have defined ‘Salmonella’, ‘Listeria’, and ‘Ecoli’. Once bacteria have been successfully cultivated and are ready for storage, a descendent ‘Frozen Stock’ item is created. When multiple single colonies of the same bacterial strains are grown, or sub-cultivated, each results in an independent ‘Frozen Stock’. ‘Frozen Stocks’ are the parents of ‘DNA’, which includes information on DNA concentration and volume. When DNA stocks are changed, e.g. after normalization to a different concentration, a new child ‘DNA’ item is generated. The ‘DNA’ that is used for PCR is the parent of a ‘PCR product’, which includes reaction-specific information, including the gene fragment being amplified. ‘PCR products’ are the parents of one or more ‘Sequencing reaction’. ‘PCR products’ and ‘Sequencing reactions’ contain links to ‘Oligonucleotide’ and ‘Working stock’, which are in a separate hierarchy (Fig. 1b).

These procedures generated >200,000 ‘items’ (Table 1), excluding records reflecting the history of changed items. Most of the >25,000 bacterial strains in our collection are currently either *S. enterica* or *Listeria monocytogenes* [19]. However the system is flexible, and was designed to include other species as well as DNA samples which were obtained without their corresponding bacterial strains.

**Table 1:** Number of Items in sample management system.

Item type	Number of Items*
Bacteria	25,599
Original bacterial tubes	39,442
Frozen Stock	35,255
DNA	14,870
Normalized DNA	5,959
PCR product	59,870
Sequencing reaction	26,950
Oligonucleotide	1,380
Working Stock	325
Total	209,650

* As of March 2012.

### Bar-coding

We implemented a system that results in reliable tracking of all physical items and experimental procedures by using bar-codes on all microwell plates and on all tubes containing bacteria or DNA. These 2-D bar-coded and screw-capped tubes (‘2D tubes’) are stored in 1-D bar-coded 96-well racks (Fig. 1c). The absence of all coding information other than bar-codes obliges the user to use 1-D and 2-D bar-code readers, which are installed in all laboratories and are supported by a series of custom designed, user-friendly, graphical user interfaces (GUIs), all of which are written in Python, or within the user interfaces to the robotic systems (Overlord, WinPrep).

A generic, six level hierarchy of locations is implemented within ItemTracker (Fig. 1c). We assigned location designations for each position in a tube rack or microwell plate, each shelf within a storage rack, each storage rack within a container (freezers, refrigerators, shelves), and each container within a room, as illustrated at the bottom of Fig. 1c. These locations are assigned to each tube and rack by the various GUIs we have implemented. For example, one GUI in the freezer room (Table S2 Script A10) not only provides information on the contents of scanned tubes and their rack ID, but also highlights all discrepancies between the recorded rack locations *versus* their current locations (Fig. S2A). This feature permits the user-friendly reorganization of tubes into new racks.

### LIMS

We integrated the SMS and bar-coding information into a LIMS through additional GUIs that link that data with a commercial genotyping and sequence analysis software (Bionumerics) (Fig. 1a). Detailed strain information, sequence traces and results of SNP typing are stored in Bionumerics, which also can be used to manually evaluate traces, assemble contigs, assign MLST allele numbers and perform population genetic and phylogenetic analyzes. Our scripts automatically import sequence traces and evaluate trace assemblies (Script D8, D10). Where manual intervention is required because sequence traces failed or were of low quality, users can select traces for repetition (Script D10, Fig. S3b), thereby storing their identifiers in CherryPicking, a third database. Another GUI (Script A6, Fig. S3a) allows viewing data from the CherryPicking database which is collated with the SMS locations of the corresponding, most recent normalized DNA. As samples move through our pipeline, our scripts update the LIMS with information on every item and experiment.

Our LIMS has been continuously developed over a four year period. During its first two years of operation, we encountered a few strain mix-ups through human errors that were not prevented by our scripts, leading to the implementation of additional data checkpoints. Furthermore, we became even more conscious about the importance of guiding users, and preventing false decisions during the remainder of the development phase.

### General Concepts of the Pipeline

**Figure 2:**
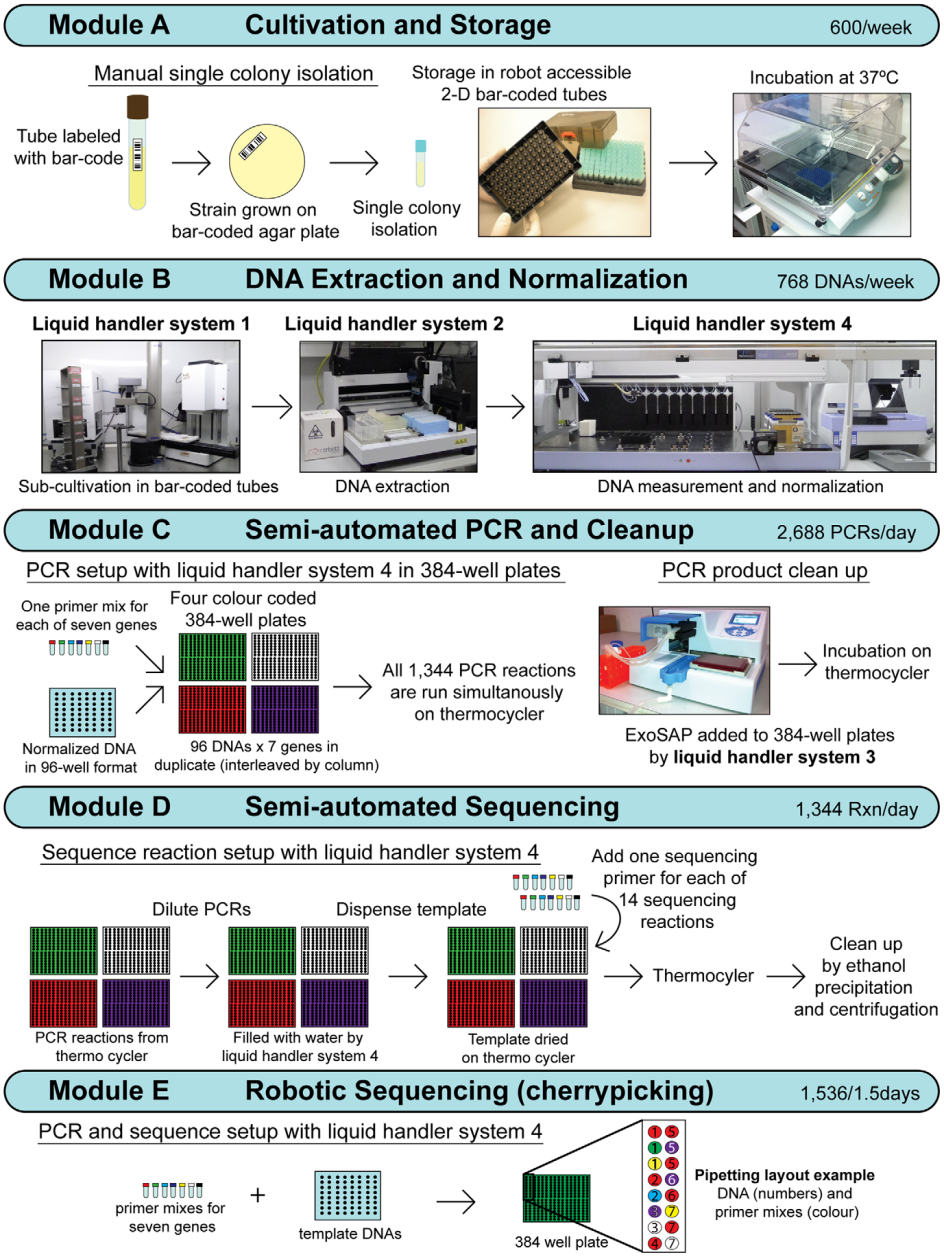
Overview of five discrete modules within the pipeline with indications of their throughput and principles. This figure includes photographs of the four liquid handler systems, LHS1-4, which are shown in greater detail in Fig. S1. Still other details on LHS1 are in Fig. 3A and File S2. Additional details on Module E are in Fig. 5. GUIs used in Modules A and D/E are in Figs. S2 and S3, respectively.

Our pipeline facilitates all steps of bacterial genotyping from the arrival of bacterial strains in the laboratory to the evaluation of sequence traces. It consists of a series of sequential modular steps, Modules A through E, each of which feature comprehensive LIMS integration (Fig. 2). Each module performs a discrete block of tasks that results in a storable product, e.g. Module A consists of cultivating and storing bacteria in freezers. Unlike assembly lines which need to be run continuously for maximal efficiency, modularisation has advantages for a microbiology laboratory, which needs to be flexible to cope with changing demands. For example, over long periods of time, Module A was run continuously while subsequent modules were being developed by other scientists. These modules can also be adapted to use alternate sources of material. For instance, Module E performs automated sequencing starting with 2D tubes containing DNA but could be used for DNAs provided by collaborators, once these were transferred into 2D tubes and their information entered in the LIMS.

We automated large parts of the pipeline through the use of robotic liquid handler systems, which result in higher throughput and reduced costs. These robots and the LIMS minimise human error, perform time-consuming mundane work and free scientists to focus on their research. However, we continue to perform certain parts of the pipeline manually. These manual steps could potentially also be automated [1], but the costs for development and equipment may be prohibitive for a small microbiology group. The most labour intensive manual work we perform is streaking to single colonies, but even this is facilitated by automated data entry in the LIMS and support by the GUIs. Other manual steps include storing racks in freezers; reorganizing tubes in new racks; moving plates and tube racks between stations and thermocyclers; sealing PCR plates; and centrifugation, but these are not particularly onerous. Our pragmatic balance between manual work and robotic automation resulted in low cost and medium throughput (Table 2). It has been well received by our technical staff, with the remaining manual steps providing a sense of involvement in data production plus visual feedback on the success at intermediate steps within the pipeline.

**Table 2:** Time and costing of pipeline for processing 96 strains.

Module	Total time(hours)	Hands on time(hours)	Cost of consumables per strain (€)
A. Cultivation and Storage			
Data input into databases	0.5	0.5	
Original tube labelling	0.5	0.5	
Streaking from tube onto plate	1.5	1.5	0.37
Incubation	Overnight	0	
Single colony into 2-D bar-coded 1.4 ml tube	1.5	1.5	0.43
Incubation	Overnight	0	
Sub total	3 days	4	0.80
B. DNA extraction and normalization			
Sub-culturing and transfer into deep well plate	1	0.5	0.59
Incubation	Overnight	0	
DNA extraction	2.5	1	1.37^1^
Normalization of DNA concentrations	1.5	0.5	0.73^1^
Sub total	2 days	2	2.69
C. Semi- automated PCR and Cleanup (4×384)			
PCR Setup	2.5	0.5	0.87
Thermocycler	2	0	
PCR cleanup	1	0.25	0.99
Sub total	5.5	0.75	1.86^2^
D. Semi- automated sequencing (4×384)			
PCR product dilution	1.5	0.25	0.12
Template transfer (incl. drying pellet)	1.5	0.25	0.12
Sequence reaction setup	1	0.25	2.14
Thermocycler	2	0	
Sequencing cleanup	2.5	0.5	
Automated sequencing	4 days^3^	0	10.76
Evaluation of traces (ABI)	7	7	
Sub total	4 days	8	13.14^2^
E. Robotic Sequencing (1×384)			
Rearrange DNAs into one rack	0.5	0.5	
PCR Setup	1	0.5	
Thermocycler	2	0	
PCR Clean up	1	0.25	
PCR product dilution	0.5	0.25	
Template transfer (incl. drying pellet)	0.5	0.25	
Sequence reaction setup	0.5	0.25	
Thermocycler	2	0	
Sequencing clean up	2.5	0.5	
Automated sequencing	4 days^3^	0	
Evaluation of traces (ABI)	1.5	1.5	
Total	14 days	19	18.49

1 Costing includes €0.40 per 2-D bar-coded tube.

2 Costing includes a repeat rate of 10%.

3 Sequencing is performed externally, which usually results in a delay of 3–5 days before sequencing traces are available.

In the following sections, we describe each of the five modules of the pipeline.

### Module A: Cultivation and Storage

We implemented a pen and paper free environment for microbiological cultivation and storage of bacteria, in order to avoid problems associated with unstructured data management and legibility of handwritten labels. Critically, we first digitise all strain information in a standard format with the help of Script A2, which creates new ‘Bacteria’ items with the appropriate ‘StrainID’ and a child ‘Salmonella’/’Listeria’/’Ecoli’ item with a unique ‘ItemName’.

Duplicate labels are generated and printed with human-readable text (‘StrainID’, ‘ItemName’) plus a bar-code (‘ItemName’) (Script A7). The use of our own Python script freed us to print these labels in any desired order, whereas ItemTracker provided only limited flexibility. The labels are affixed to the original container and a fresh agar plate (Fig. 2). The bacteria are manually streaked to single colonies in a safety cabinet while running a GUI (Script A9, Fig. S2b). If the paired bar-codes differ, this script issues a distinctive error tone. When the correct bar-coded item cannot be located at this juncture, this item can be excluded. The user then continues with the next set of matching bar-codes. When matched bar-codes are scanned the script updates the status in the SMS for these bar-codes. Audible error tones allow the user to focus on manual work and not on the computer screen. The agar plates are then incubated overnight for bacterial growth.

After incubation of plates, 1.4 ml 2D tubes containing sterile growth media are each manually inoculated with a single colony. Script A9 associates the bar-code on the agar plate with the 2-D bar-code of the tube within a newly created ‘Frozen Stock’. After incubation of the tubes, and just before freezing, the status (‘Viability F’  =  ‘growth’) and locations of 2D tubes within which bacteria grew are updated in the SMS (Script A10). Rare tubes lacking visible growth are removed from the rack and discarded, deleted from the SMS, and the viability of their parent is set to ‘no growth’. Additional strain information for bacteria that grew is now imported into Bionumerics using Scripts A3–A5, which ensure consistency with the properties of existing data entries, including style and spell-checking. Other data that have been manually transcribed into Microsoft Access [19] can also be imported into Bionumerics (Script A16).

The result of these procedures are bacterial strains in space-saving and robot-friendly 2D tubes in defined locations within our freezers (Fig. 1c), as well as the locations of all other tubes containing bacterial strains [19].

### Module B: Sub-cultivation, DNA Extraction and Normalization

**Figure 3:**
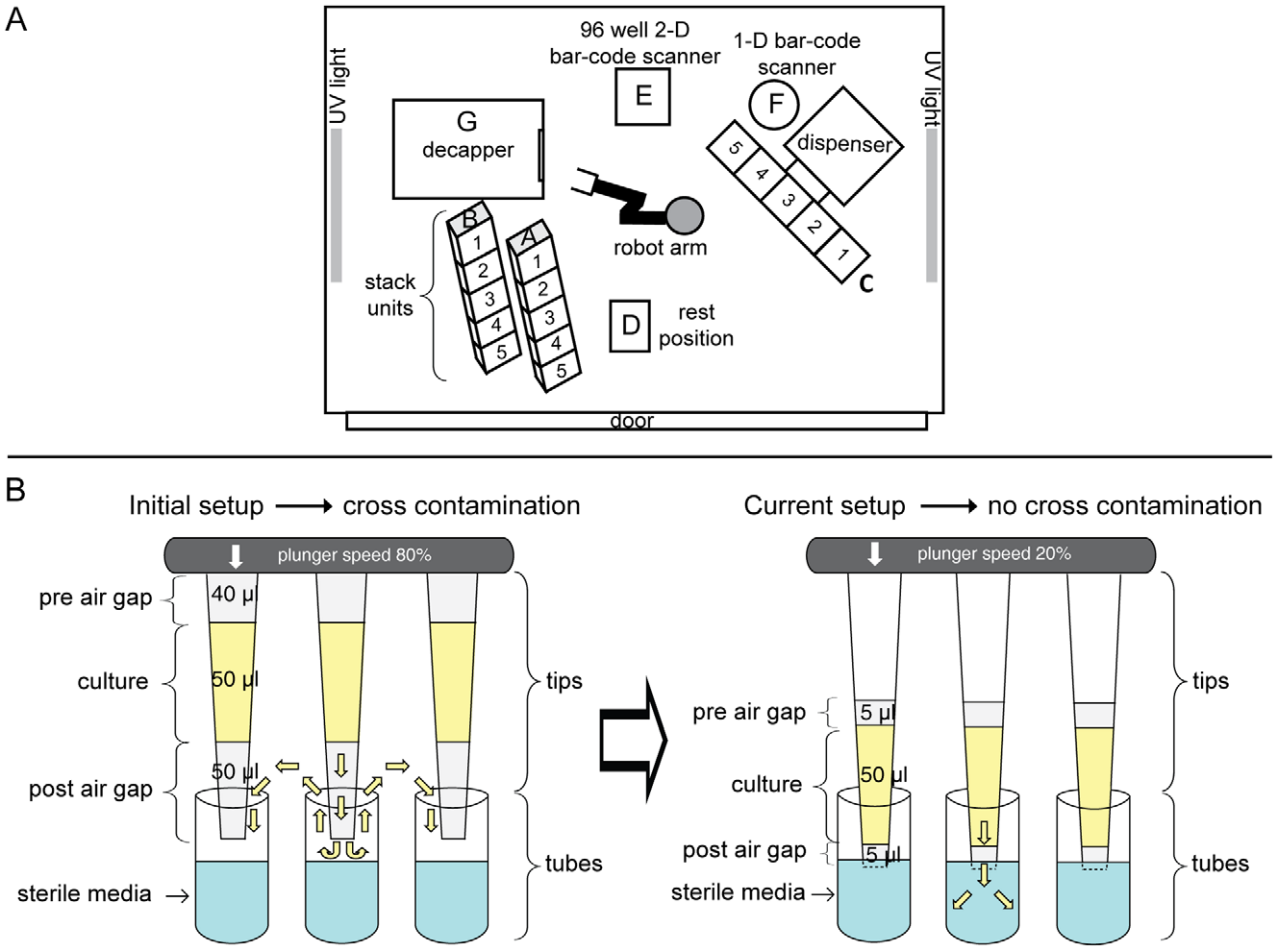
Automated sub-cultivation with LHS1. (A) Schematic layout of the equipment within the LHS1 cabinet. Positions A and B represent stack units where racks of 2D tubes and deep well plates are stored prior to operations. Position C designates the 5-position, XY-moveable stage for the 96-tip pipettor. Position D represents a rest position used for regripping of racks of 2D tubes before and after capping/decapping in position G. Positions E and F represent 2-D and 1-D bar-code readers. H is a SCARA robotic arm for moving between these positions. Further details of how LHS1 was used are presented in File S2, Figs. S6/S8 for sterile dispensing of media into 2D tubes, and S7/S9 for aseptic sub-cultivation of bacteria into 2D tubes and deep well plates. (B) Improvements in pipetting parameters (right) which eliminated aerosol formation that led to cross-contamination between cultures (left).

Module B extensively exploits the robotic fluidics provided by four liquid handler systems (LHS1-4) (Fig. 2, Fig. S1). LHS1 and LHS4 automatically scan the 1-D bar-codes of all racks and microwell plates as well as the 2-D bar-codes of tubes, and store that information in the SMS. LHS1 was custom-built for microbiological work. It consists of a class 2 safety cabinet with sterile air flow containing an automated capper/decapper, a 96-tip pipetting robot and a robotic arm (Fig. 3a). LHS1 automatically and aseptically dispenses sterile liquid media to racks of 96 2D tubes (for Modules A and B) and deep well plates (DWPs). LHS1 also aseptically dispenses cultivated bacteria to such racks and to DWPs. LHS2 is a commercial, 8-tipped robot which we used for automated extraction of 96 DNAs from such DWPs. LHS4, supported by LHS3, was used to quantify and normalize DNA, providing a replaceable, low concentration working stock for downstream procedures. LHS4 includes a pipetting robot that is capable of handling multiple pieces of labware, with eight independent, liquid-sensing tips and a gripper arm; a fluorimeter; and bar-code scanners. A manually operated capper/decapper in the vicinity is used to decap 2D tubes prior to pipetting and recap them afterwards. Because simple multi-dispense steps were slow with this syringe-based pipetting robot, we dispensed multiple reagents with LHS3, a manually-operated dispenser.

LHS1 and LHS4 are each supported by their own central GUIs written in Python (Scripts B1 and B5, respectively), each of which offers radio buttons or drop-down menus to run of the collection of other scripts and procedures that were developed for the various manipulations described below. Script B1 also calls procedures in Overlord, which is the commercial operating system used to control LHS1 while Script B5 calls procedures in WinPrep, the operating system for LHS4. The use of these GUIs provided the LIMS with the needed flexibility to handle file manipulations, update the SMS with data from scanned bar-codes, and calculate standard curves and DNA concentrations.

Module B incorporates full LIMS integration and allows the medium throughput, with negligible cross-contamination, that is required for extensive genotyping of large strain collections, such as are typical of many reference and diagnostic laboratories. This level of automated throughput has allowed three microbiologists to sub-cultivate and extract DNA from >10,000 bacterial isolates, and normalize most of those DNAs, over the last two years (Table 1).

#### Overcoming cross-contamination during sub-cultivation

We use LHS1 to sub-cultivate bacteria into 2D tubes for frozen stocks and DWPs for DNA extraction (File S2). Each rack of 2D tubes is decapped by 96 miniature screwdriver tips in an automated capper/decapper. The caps remain on the screwdriver tips until fluid has been pipetted to the tube racks by a 96-tip dispenser, at which point the tubes are re-capped. We were concerned whether cross-contamination might occur through aerosols that arose during decapping, dispensing or recapping. Initial experiments with a checkerboard arrangement of 1.4 ml 2D tubes containing bacteria and sterile media showed that heavy cross-contamination of the progeny tubes occurred during pipetting (Fig. 3). This cross-contamination was caused by multiple factors. We were able to totally eliminate contamination among ten racks of progeny tubes in checkerboard experiments by pre-wetting with sterile liquid media, use of minimal air gaps, slow plunger speeds and dispensing fluids below the liquid level. During these same experiments, one sterile, control parental 2D tube became contaminated (0.2%), much less frequent than is the case during manual inoculation [20]. This residual contamination likely arose through miniscule amounts of aerosol in the capper/decapper. Because the parental 2D tubes would already contain >10^9^ bacteria, this is unlikely to be problematic in practice. To avoid even this low residual risk, we always make a new child frozen stock when opening parental 2D tubes to inoculate DWPs, and use the most recent frozen stock for all sub-cultivations.

#### DNA extraction

**Figure 4:**
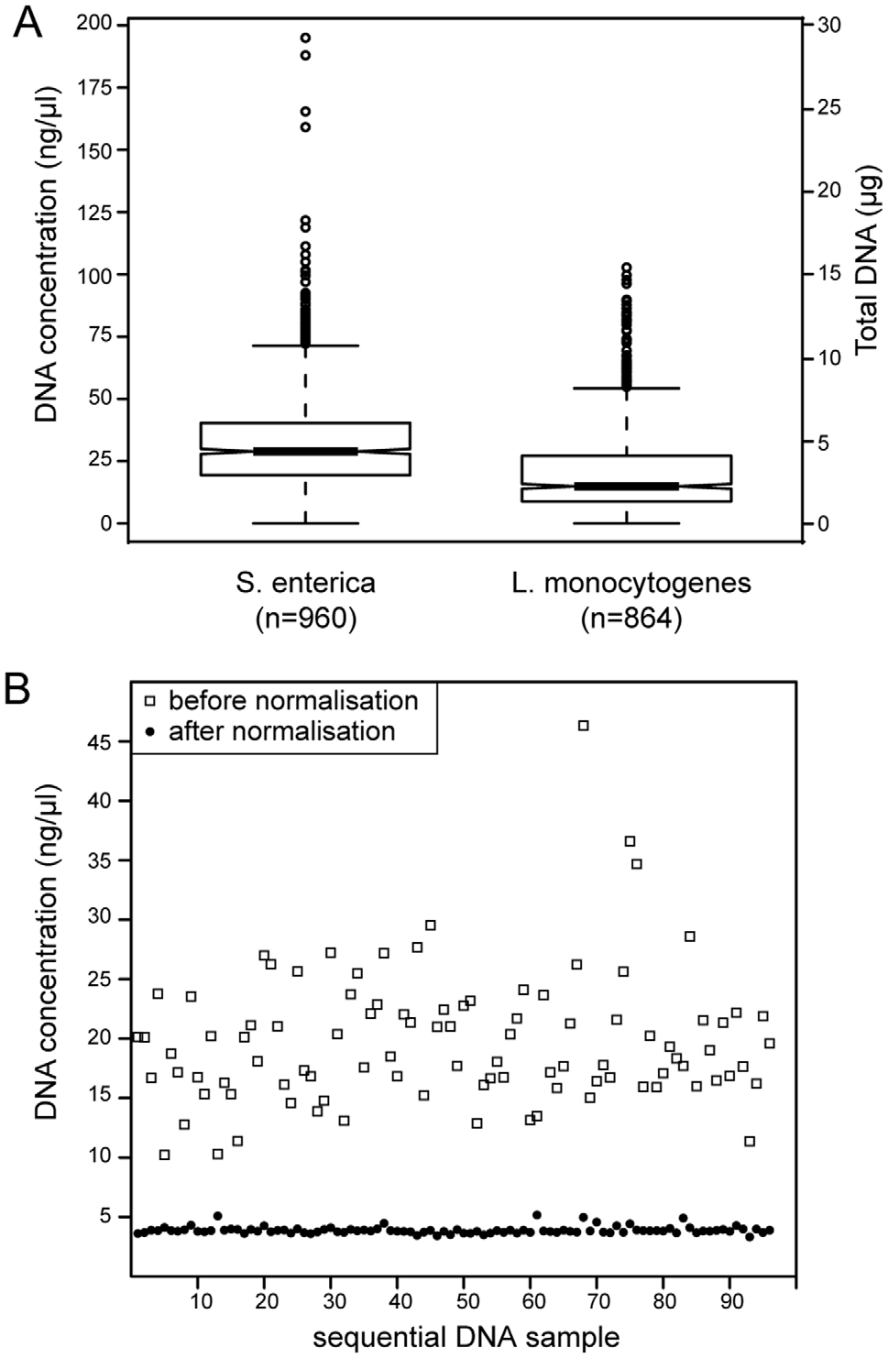
Yields of DNA after automated extraction and normalisation in Module B. (A) Notched box and whiskers plots of DNA yields after automated DNA extraction from *S. enterica* or *L*. *monocytogenes*. The notch indicates the 95% confidence estimate of the median value (central line), which splits the boxes in the second and third quartiles of the data. The first and fourth quartiles are indicated by the external horizontal lines with outliers shown by single circles. The number of samples that has been summarized is indicated in parentheses under the bacterial designations on the X axis. The left scale reflects the DNA concentration and the right scale is the total yield within the 150 µl elution volume. (B) DNA concentrations for one rack of 96 tubes of *S*. *enterica* after elution as in part (A) (empty squares), and after automated normalisation (solid circles) to 3.3 ng/µl.

Our goal was a consistent, medium throughput method for extracting at least 2 μg of DNA from bacterial cultures, which would be adequate for both genotyping and genomic sequencing. LHS2 automatically extracts DNAs from 96-well DWPs (Script B17). However, dedicated kits from the manufacturer were designed for extracting DNA from blood or tissues, and did not yield enough DNA from either *L. monocytogenes* or *S*. *enterica* because bacterial lysis was inefficient. We improved the yield by combining a bacterial DNA lysis method from a second manufacturer with a generic bind-wash-elute method [21] from a third manufacturer, followed by elution of DNA into 150 μl in 0.5 ml 2D tubes. We also identified cheaper, alternative suppliers for tips, resulting in total costs of 1.37 Euro per strain for extracting DNA (Table 2), with potential throughput of >600 DNA extractions per week. These procedures yielded a median of >2 μg of DNA from either *S. enterica* or *L. monocytogenes* (Fig. 4), with only occasional dropouts, which reflected either plugged filters due to too many cells/DNA or wells which failed to grow. These dropouts included ∼1% of the *L. monocytogenes* strains which grew so slowly that DNA isolation needed to be performed manually. Repeating the other exceptional dropouts yielded satisfactory DNA yields.

#### DNA normalization

A standard dilution of the source DNA in a new 2D tube was quantified (PicoGreen fluorescence) for quality control of DNA extractions by LHS4 with the aid of LHS3. Our scripts drive the fluorimeter, obtain the fluorescence values, generate a standard curve against known DNA concentrations, and calculate DNA concentrations of the diluted DNAs (Scripts B10–B11, B15). They also calculate a correction factor of additional DNA or diluent, which is used to robotically adjust the diluted DNA to a defined concentration (Script B11), except for DNAs where the resulting volume would be too large, in which case a message is displayed detailing the affected DNAs. Alternatively, robotic normalization of DNAs can be performed from known concentrations that have been previously recorded in the SMS (Script B16). The efficiency of these normalisations is documented in Fig. 4b.

The result of these manipulations are matched sets of one frozen sub-culture and two 2D tubes containing the quantified, extracted DNA and the normalized DNA.

### Module C – Semi-automated PCR and Cleanup

This system was developed for MLST as a reliable and rapid replacement for manual PCR and sequencing reactions. In MLST of *S. enterica* [20] or *L. monocytogenes* [22], for each of seven gene fragments, two independent PCRs, forward and reverse, are performed in order to avoid PCR errors [23]. Manual PCR reactions used 96-well microwell plates, requiring 42 thermocycler runs for each batch of 96 DNAs, 14 each for PCR, cleanup and sequencing, which was a major bottleneck. Intervening centrifugation steps presented further bottlenecks, particularly the hour-long centrifugation step used in cleaning sequencing reactions.

Switching to 384-well plates in our automated procedures improved the throughput through these bottlenecks by 3.5fold, and also reduced reagent costs due to lower reaction volumes. A further reduction in consumable costs was achieved by using only fixed tips in Modules C–E. We used a bleach washing step after dispensing each distinct DNA during PCR setup to help prevent carry over of DNA between wells. The rationale of this approach derived from communication with the manufacturers of LHS4, who described the use of bleach in system liquid by other customers to help prevent contamination. A bleach solution has also been used to remove surface DNA contamination from bones and teeth [24].To reduce pipette motion, the number of wash steps and potential cross-contamination, we interleaved two gene fragments per plate by columns, placing each DNA in a quadrant of four adjacent wells. Given our experience in Module B, DNA was dispensed below the liquid level to help prevent aerosol formation. Under these conditions, checkerboard experiments did not result in any false PCR products in negative controls. In practise, we did not observe any contradictory peaks between paired sequence traces from over 25,000 sequencing reactions. However, we note that we do not use a bleach wash step in Module E (described below), with no obvious detriment. Other laboratories interested in our pipeline may wish to experiment with removing the bleach wash step in Module C.

In the standard PCR setup, reactions were performed in four bar-coded and color-coded 384-well PCR plates (Fig. 2; Script C5). Consistent color-coding was used to facilitate user recognition of parent-child relationships between plates in subsequent procedures. Module C uses WinPrep to show a graphic of the LHS4 deck, including color-coded locations for each microwell plate. LSH4 then scans all labware bar-codes, including the bar-codes of 2D tubes containing template DNA. A second GUI (Script C4) allows the user to select the pair of gene fragments and suitable primer working solutions for each plate. That GUI also indicates where to place tubes of each primer master mix in the cooling thermoshaker and their required volume. Novel PCR products are then created in the SMS, including information about gene fragments, primer working stocks, *etc*. (Scripts C1–C3).

LHS4 was used to first dispense appropriate primer master mixes to each of the seven gene positions in duplicate, and then DNA to all 14 wells. Subsequently, a standard mix of water, buffer and *Taq* polymerase is added using LHS3. The plates are sealed, centrifuged and thermocycled. Residual dNTPs and primers are removed from the PCR products with ExoSAP, which was made more cost effective by reducing the concentrations below those recommended by the manufacturer. Using Module C, we are able to perform 2,688 PCR and cleanup reactions (two sets of 96 normalized DNAs) within one day (Fig. 2, Table 2). The cleaned products can be stored frozen for subsequent processing, or used immediately in Module D.

### Module D – Semi-automated Sequencing

Based on the bar-codes of the microwell plates from Module C, information is collated from the SMS about the sources of the PCR products (Script D3). LHS4 dilutes the cleaned PCR products in new, color-coded 384-well plates containing water previously dispensed with LHS3 (Script D5), and updates the SMS with new child ‘PCR products’ including the collated information, whose locations refer to the bar-codes for the new plates (Script D1). These plates are manually sealed, mixed by vortexing and centrifuged because mixing would be much slower with LHS4, and might create air bubbles. The mixed dilutions are then returned to the LHS4 deck for dispensing primer working solutions. Primer working solutions that are appropriate for each gene fragment according to the information from the new ‘PCR products’ are presented to the user via drop down lists (Script D2). The diluted PCR products are dispensed to a third set of color-coded 384-well plates (Script D6), again similarly updating the SMS. These DNA templates are dried to reduce the volumes of sequencing reagents. Concurrently, we routinely perform manual gel electrophoresis on eight PCR reactions for each gene fragment to test PCR quality before proceeding.

In the next step, the information on the sequencing reactions for the bar-coded plates is retrieved from the SMS and the user is instructed where to place the vials containing the selected sequencing primers (Script D4). LHS4 then dispenses the sequencing primers that are appropriate to each well (Script D7), and updates the SMS. BigDye is added to each sequencing reaction with LHS3 and sequencing is performed on a thermocycler. The sequencing reactions are cleaned up by ethanol precipitation in the 384-well plate. Module D produces 1,344 ethanol-precipitated sequencing products per day.

The plates containing dried sequencing reaction pellets are shipped to an external sequencing service where the reactions are re-suspended and run on a sequencing machine. The resulting trace files are named using information files that were previously generated by LHS4 (Script D2). After downloading the trace files, these informative names are used for automated import of traces into Bionumerics (Script D8), followed by assessment of the quality of the sequence assemblies (Script D10). Our scripts automatically approve assemblies with two or more traces that meet two criteria: (i) both trimming sites must be found and (ii) all positions must be unambiguously supported by traces from two or more independent PCRs. Should these criteria not be met, Script D10 pauses, and opens an assembly window to allow manual edits. 2–10% of traces need to be repeated due to poor PCR products or poor sequencing, and in those cases, the GUI supports recording the traces that need to be repeated (Fig. S3b), which are collated in the Cherrypicking database.

Including the four day delay before sequencing traces are available for downloading, our pipeline allows MLST of 96 strains, including trace evaluation to be performed within 14 days after receiving bacterial cultures, at a per strain total cost of under 20 Euro (Table 2).

### Robotic Sequencing – Module E

Module E was designed to facilitate repeating sequences which had failed. However, it has the potential to become a general tool for varied and automated design of complex sequencing experiments for any combination of DNAs that is included in the CherryPicking database. To this end, optimal combinations of 96 DNAs that will best fill one or two 384-well plates for efficient downstream processing are generated from the Cherrypicking database for user-defined species and combinations of genes (Script E11). The locations of these optimal sets of DNAs according to the SMS are also provided, which allows rapid assembly of a rack of DNAs for robotic sequencing.

**Figure 5:**
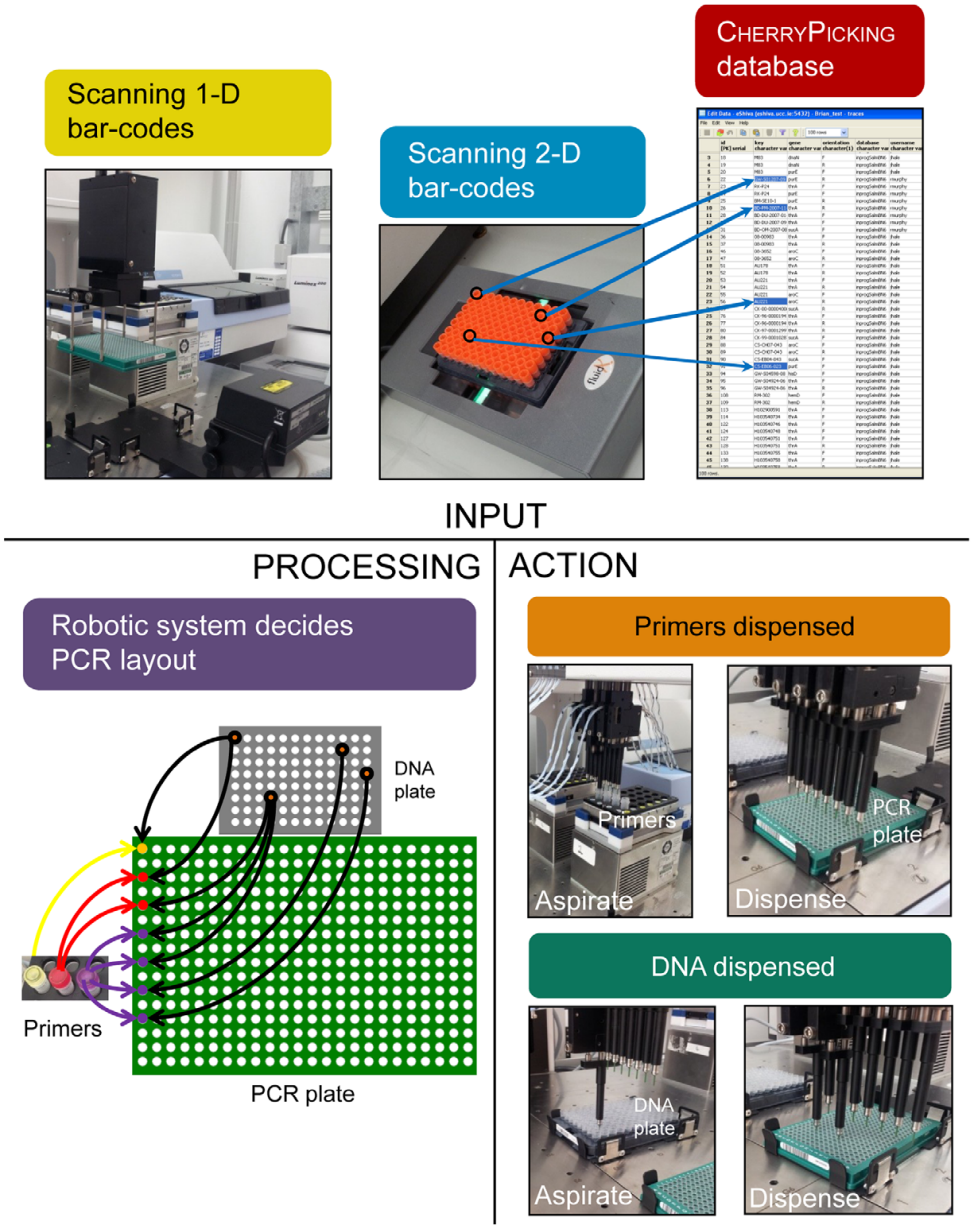
Overview of robotic sequencing in Module E. Top. 1-D and 2-D bar-codes of DNA tubes and 384-well plates are scanned, and information for genes sequencing, including orientation, is collated from the Cherrypicking database for the scanned DNA tubes. Bottom. Left: a pipetting layout which optimises pipetting efficiency is calculated by Script E7 for one to two 384-well plates. Color-coded arrows indicate different genes for each DNA which are to be sequenced. Right: LHS4 dispenses primer mixes from 2 ml tubes in a cooled thermoshaker to a 384-well plate, followed by DNA.

The basic protocol used in Module E is similar to those in modules C and D, but Module E is far more sophisticated in its interactions with the LIMS than are these other modules (Fig. 5). PCR setup is initiated after placing a DNA rack containing 96 2D tubes on the LHS4 deck (Script E7). For greater flexibility, this rack need not include DNAs that were previously selected by Script E11. Instead, information on the DNAs in the rack is independently obtained from the SMS and the Cherrypicking database (Script E2), and used to create a list of gene fragments that need to be repeated for those DNAs. The user chooses the genes to be sequenced and the primer working solutions for those sequences from custom drop down lists. Once again, the script instructs the user on the required volumes of primer master mixes, and where to place those tubes. The script then calculates a layout of wells on the 384-well plate(s) that is optimised for pipetting the selected DNAs, gene fragments, and sequencing orientations. PCR reactions are then dispensed according to that layout and new PCR products with the appropriate information are created in the SMS (Script E7). Subsequently, PCR products are cleaned and diluted as in Modules C and D, except that the script is designed for only one or two 384-well plates (Scripts E3, E8). The sequencing reaction setup is also similar to Module D, except for the pipetting layout (Scripts E4–E5) and minor pipetting details (Scripts E9–E10). Traces are also evaluated as in Module D.

Manual cherrypicking is one of the most onerous and error prone tasks in the production of sequence data, and a major bottleneck for completing MLST. It is here that automation and the LIMS combine to produce a true robotic system that enormously accelerates the task of identifying and reorganizing tubes of DNA for repetition, eliminates any need to replace those tubes in their original locations, and readily deals with any complicated layout of sequencing reactions that is required by the selected DNAs.

## Discussion

Considerable progress has been previously made on automating individual elements of the pipeline described here. Automated, high-throughput DNA extraction and sequencing was implemented in multiple core sequencing laboratories soon after the beginnings of the genomic revolution in the mid-1990’s [25], and aspects of bacterial genotyping were automated in some laboratories soon thereafter [26]–[28]. Bacterial diagnostics is increasingly being automated [10] and large-scale, robotically-assisted mutation discovery in human biobanks has been integrated into a LIMS [29], [30]. Conversely, we are also aware of microbiological laboratories which have reverted to manual work because robotic fluidics were too complicated, inflexible, or prone to user errors; as well as laboratories where humans are turned into automatons in a tedious, assembly line. Here, we describe a novel, integrated LIMS-based pipeline for medium-throughput bacterial genotyping, spanning the period from the arrival of bacterial cultures through to sequence evaluation. Our pipeline is user-friendly due to multiple graphical user interfaces (GUIs), and is highly regarded by our technical staff for its convenience and reliability.

### Throughput, Costs and Security

Our LIMS has allowed our small team of scientists to handle >200,000 items in the four years since we began to develop this pipeline. The pipeline saves time and costs (Table 2), with a potential throughput of up to 384 bacterial strains for one person in ten working days, including cultivation through to dispatch of sequencing reactions. Sequence trace evaluation and assignment to MLST alleles takes an additional 1–2 days per 96 strains. The combined personnel and consumables costs for MLST with this pipeline are currently under €25 per strain. This required an initial investment in robotic equipment and commercial software of nearly €350,000 (plus VAT). However, without the assistance of the pipeline, comparable costs would have been incurred for additional personnel to achieve the same throughput. We designate our throughput as medium by comparison with the true high throughput that can be found in industrial settings and core facilities.

A great advantage of the pipeline is that all tubes and microwell plates can be readily and unambiguously located. Manual movement of racks to unrecorded locations remains a potential danger, which is diminished through strict rules on recording all rack movements in the LIMS. Misplacing storage tubes is unlikely because our tubes are unlabelled except for their 2-D bar-codes, obliging users to use bar-code readers, which automatically register novel locations in the LIMS. A second advantage is the parent-child hierarchy we implemented, which provides a direct bidirectional link from sequence traces through DNA samples to the original bacteria. Such hierarchies are difficult to implement with spreadsheet-like formats. Thirdly, the LIMS preserves knowledge in digital form that would otherwise be stored in lab-books, depend on the memory of permanent lab staff and/or be endangered by changes in lab personnel [11]. Finally, our system is auditable because it records all data changes, as well as their dates and who made the changes.

The robot-friendly 2D tubes were also suitable for manual manipulations assisted by single-tube 2-D bar-code readers and capper/decappers. Our results showed that these tubes supported efficient and reliable growth, even after long-term storage of frozen bacteria. They can be rearranged without removing their seals, which is preferable to the common practice of storing DNAs in microwell plates. LHS1 allowed reliable, automated sub-cultivation in 2D tubes, even for organisms which are particularly prone to cross-contamination, such as *S. enterica* [20]. LHS1 also potentially facilitates the rapid replication of thousands of strains for distribution to other laboratories.

### Rationale of Our LIMS Implementation

The backend of our pipeline is a mixture of commercial, multi-user programs (ItemTracker, Bionumerics, Microsoft SQL) and robot-specific handling software (Overlord, WinPrep) with an open source SQL database (PostgreSQL), all glued together with extensive scripting in a generic programming language (Python). This mixture reflects a deliberate decision to purchase commercial programs that provide technically sophisticated interfaces, database structures and functionality at a cost that is affordable for a small research grant. The programs needed only minor modifications to meet our requirements whereas implementing all these features ourselves with open source software would have required much greater effort. For example, Bionumerics provides a very comfortable graphical interface to population genetic and phylogenetic data mining and presentation, which would be difficult to design *de novo.* ItemTracker provides a reliable sample management system (SMS) with a functional, flexible underlying data structure, including traceability and a GUI. Similar to the experience of others [31], our decision resulted in rapid implementation of a useable pipeline which suited our purposes, and allowed us to focus on optimizing experimental details and robotics. However, we note that open source SMS and LIMS have been described [29], [30], [32], [33], which could potentially replace the commercial SMS that we used here. Similarly, it would be relatively easy to use relational database servers other than Microsoft SQL and/or PostgreSQL, because Python includes broad support for various dialects of SQL (Structured Query Language).

It might be argued that a commercial, custom-designed monolithic LIMS would be more efficient and reliable than our home-made LIMS. However, these are considerably more expensive than the solution we implemented, and often continue to require modifications that can only be implemented by professional IT specialists. Our decentralised LIMS allowed us to integrate new flexibility over time without external assistance, such as CherryPicking, which was developed two years after the other components. Our LIMS is underpinned by Python, which is readily accessible, user-friendly and suitable for team programming. While we have full confidence in our LIMS, we appreciate that third party validation would be required before all or part of the LIMS could be implemented in a clinical environment. Although they previously had only limited programming experience, the two scientists primarily responsible for the pipeline (BOF, JKH) rapidly became sufficiently proficient in programming in Python that they were able to manipulate data, create drivers for pipeline equipment, interface with database servers and design GUIs. Other non-programmers in our group have become moderately competent in Python in a reasonably short period of time, and routinely use it for manipulation of large amounts of data. These are important skills for any modern biologist. While this pipeline was developed by biologists, prior experience in bio-engineering and programming would probably have expedited pipeline establishment.

### Pipeline Development

Given the extensive details on methodology, equipment and scripting that we provide here, it should be possible for a motivated team of biologists to quickly re-create all or part of our pipeline. Only the robotic parameters are instrument specific, and they should be readily transferrable to other robots with the assistance of their manufacturers. In general, we would recommend initially creating a robust and expandable data structure for the LIMS, and then integrating the equipment into the pipeline in a modular fashion. In addition to the benefits of modularity already described above, the modular approach permits users who are reticent about robotic approaches to become accustomed to their benefits and the style of working with robotic modules. For example, working with Module A, which fully integrates 2D tubes, LHS1 and GUIs with extensive hands-on manipulations, familiarised our staff with a robotic approach sufficiently that they welcomed the introduction of subsequent modules which reduced manual processing. The sequential development of individual modules also provided crucial feedback from the users, which informed the development of subsequent modules.

We believe that automation of mundane laboratory work should not eliminate jobs for lab personnel, but with time, will lead to shifts in their work roles, including a greater degree of multi-disciplinarity. The staff who are responsible for operating the pipeline feel increased responsibility for the final results, including a sense of ownership, at least partially because of its speed and scale. Our group now has a greater proportion of lab personnel who perform data analyzes. In the future, we anticipate that we will be better able to develop new assays that are facilitated by automation, especially because robots represent valuable tools for experimentation.

### Summary

Our robotic pipeline and LIMS ensures complete traceability, minimises human errors and facilitates rapid and efficient sub-cultivation, DNA extraction, DNA normalization and genotyping by MLST. A Luminex 200 analyzer is situated next to the LHS4, within reach of the gripper arm, which could be used for automated CRISPR typing [34] or SNP typing [35] of *S. enterica*. The pipeline could readily be expanded to include still other genotyping methods that lend themselves to automation. We anticipate that further developments of this system will facilitate preparing samples for automated genome sequencing of 1000’s of bacterial strains, possibly the ultimate form of bacterial genotyping, and which will probably become increasingly essential for tracking developments in antimicrobial resistance and for monitoring within hospital environments.

## Supporting Information

Figure S1 **Components of LHS1 (A), LHS2 (B), LHS3 (C), and LHS4 (D).**
(PDF)

Figure S2 **Screenshots of the two primary GUI Scripts used in Module A.** (A) Script A10 shows the contents of 2D tubes after bar-code scanning. 96 square buttons show the contents of ‘StrainID’ and ‘ItemName’ for each 2D tube. These are color-coded according to five categories, three of which indicate discrepancies between the tubes scanned and data in the SMS. An x symbol in a button indicates that no tube was present at that position. Clicking on one of the 96 square buttons opens a small sub-window with additional information from the SMS for the scanned bar-code and the rack location. Nine other rectangular buttons are included at the top, two of which are color-coded. Clicking on the colored button labelled “Remove/delete items” opens a vertical list of all discrepant tubes. Radio buttons associated with each tube facilitate choosing tubes to be deleted, or whose location should be deleted. “Move items” allows updating tube locations in the SMS with their current locations in this rack. The SMS also stores information on items that are “selected” by Script A6. Such selected items are colored green when “Show selected Items” is clicked. Similarly, “Show status of FrozenStock” distinguishes between the status “Confirmed” (green) and “Contaminated” (red). (B) Script A9 is used during manual microbiology. It creates new items and updaties existing linked items in the SMS. Each button offers the opportunity to compare the results of one or two bar-codes with one or two other bar-codes during various manipulations of bacteria on plates, in shipping containers or 2D tubes during transfer of material, sub-cultivation or DNA extraction.
(PDF)

Figure S3 **Screenshot of Scripts that link Bionumerics with the SMS and CherryPicking databases.** (A) Script A6 shows the properties of individual stab cultures as well as 2D tubes containing frozen stocks or DNAs within the SMS. This window initially shows bacterial strains that were selected in Bionumerics prior to running the script. The contents of the window can be changed by clicking on buttons at the top left: “Get All Selected” retrieves items in the Bionumerics database which were previously selected in the SMS by the user; “CherryPicking” displays all DNAs of strains in the Bionumerics database which are listed in the CherryPicking database with the status “Repeat”. Left panel: Tree-like representation of the parent-child hierarchy in the SMS, including selected fixed properties, such as ‘viability F’ or ‘DNA concentration’. Items without a location in the SMS are labelled in red, while items that were previously selected are marked in green. Ticked items can be selected/deselected through the Update button at the lower left. Right Panel: Additional freely selectable information from Bionumerics and the ItemTracker SMS for the highlighted item in the left panel. The sources of this information are chosen with the buttons ‘ItemTracker’ and ‘Bionumerics’ at the lower right. Bottom panel: Log of changes in selection status. (B) Script D10 script automatically attempts to complete all unfinished contigs in Bionumerics. For contigs containing two or more traces, it attempts to assign both trimming sites and checks whether each nucleotide between those trimming sites is covered by at least two traces, and that there are no discrepancies between traces. Contigs that meet these criteria are saved as finished in Bionumerics. Otherwise an assembly window is opened (not shown) as well as the control panel shown here, to give the user an opportunity to manually resolve problems or choose traces which need to be repeated. For traces that need to be repeated, the user can specify whether the forward and/or reverse traces need repetition (lower left), and this information is stored in the CherryPicking database.
(PDF)

Figure S4 **SQL tables and their relation implemented in ItemTracker.** Only tables and columns relevant for PYTHON scripts are shown.
(TIF)

Figure S5 **Cabinet layout of LHS1.**
(TIF)

Figure S6 **Overview of the Overlord procedure to dispense sterile media.** Upper right- positions within cabinet. Lower right- tube racks are stored in the stack units such that they can be removed by the robotic arm. However, the possible gripping positions are incompatible with placing the rack in the capper/decapper. Therefore, each transfer from stack unit to the capper/decapper, and vice versa, requires a regripping step which is performed at position D.
(TIF)

Figure S7 **Bacterial sub-cultivation and preparation for DNA extraction.** Left: Flow chart for scripts and procedures used. Dispenser protocols include pre-defined positions for pauses, which are triggered sequentially by Overlord protocols. Colors indicate the positions in the cabinet. Upper right: Cabinet layout, color-coded as in flow chart. Right: Procedures repeated every time tube rack barcodes are scanned (left) or tube racks are decapped and capped (right). After decapping, caps remain on screwdriver tips until recapping of the same tube rack. User input at beginning via GUI includes choice of procedure “subculture” or “DNA extraction plus subculture”, number of DNA extractions (0–2) and volumes in parent rack, child rack and deep well plate.
(TIF)

Figure S8 **Flow chart of sequential procedures used for dispensing sterile media into tube racks.** Various volumes can be dispensed into variable numbers of tube racks. Blue rectangles- dispenser procedures; [Step1.Racks]- variable for total numbers of tube racks; [CurrentPlate]- variable iterating from 1 to [Step1.Racks]
(TIF)

Figure S9 **Logical relationship of procedures used to subculture bacteria.** Procedure starts with Python Script B1 (top left) calling Overlord procedure LHS1-64 followed Python Script B2 or B3 and Overlord procedure LHS1-57. Dispenser procedures are highlighted in blue. [SourceVolume] - Variable for volume of bacterial culture in parent tubes; [DestVolume]- Variable for volume of sterile media in child tube rack; [DWP Volume]- Variable for volume of sterile media dispensed into deep well plate; [CurrentPlate]- Variable iterting from 1–2.
(TIF)

Table S1 **Columnnames in table “traces” of cherrypicking database.**
(DOCX)

Table S2 **Scripts developed for the pipeline.**
(DOCX)

Table S3 **Common, predefined properties that apply to all items.**
(DOCX)

Table S4 **ItemTypes and ItemProperties defined by administrator.**
(DOCX)

Table S5 **Purpose of Field rules that were applied to ItemProperties.**
(DOCX)

Table S6 **Field rules settings in ItemTracker.**
(DOCX)

Table S7 **General Overlord files and visual basic scripts used by LHS1 procedures.**
(DOCX)

Table S8 **Parameters for filling tube racks.**
(DOCX)

Table S9 **Overlord and Apricot files used by LHS1 for dispensing sterile media.**
(DOCX)

Table S10 **Overlord files used by LHS1 for sub-culturing bacterial cultures.**
(DOCX)

Table S11 **Visual basic scripts used by Overlord for sub-culturing bacteria.**
(DOCX)

Table S12 **Protocols used for configuring the dispenser of LHS1 for various labware and volumes for the sub-culturing procedure.**
(DOCX)

File S1 **Configuration of ItemTracker.**
(DOCX)

File S2 **Setup of liquid handling system 1 (LHS1).**
(DOCX)
