# Supplementary figures and images for: Transforming Microbial Genotyping: A Robotic Pipeline for Genotyping Bacterial Strains
Source: PLoS One. 2012 Oct 29;7(10):e48022. doi: 10.1371/journal.pone.0048022 (PMC3483277; doi:10.1371/journal.pone.0048022)

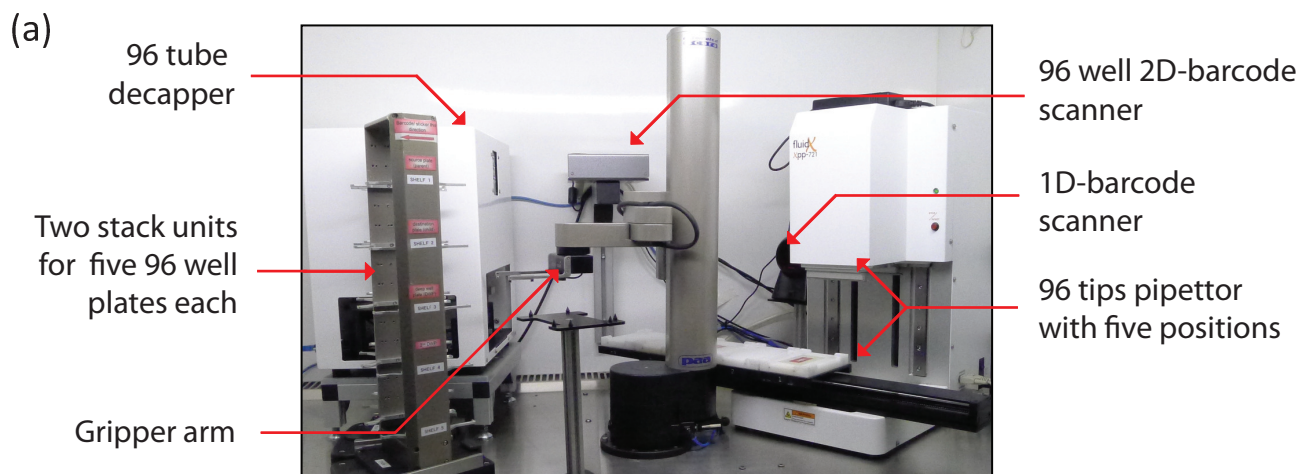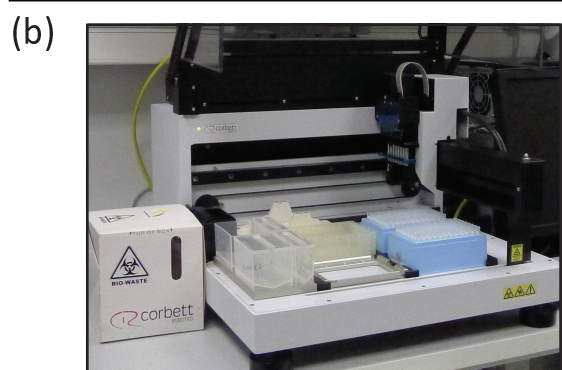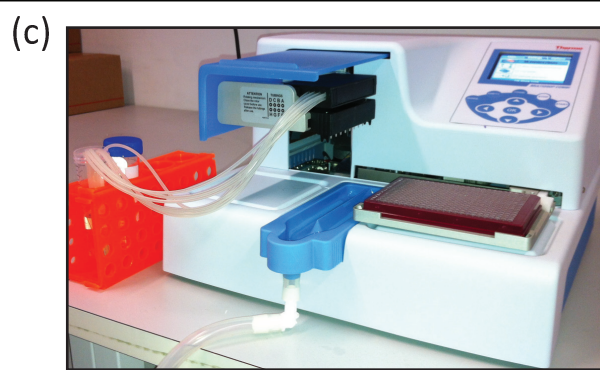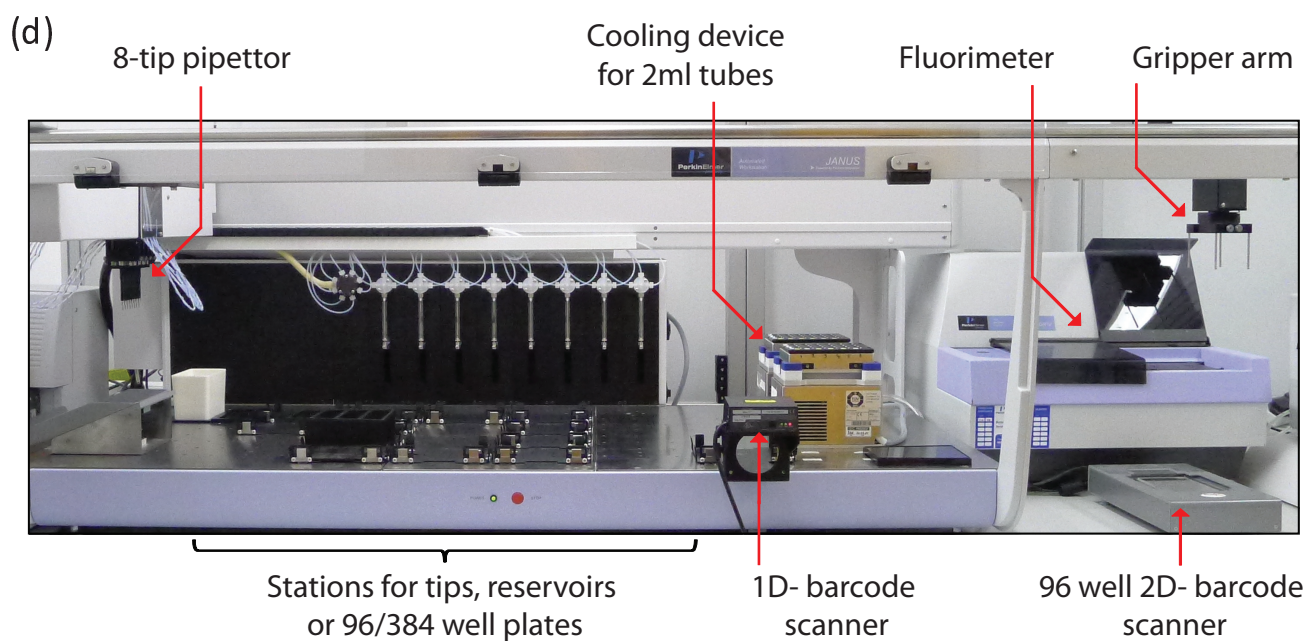

**Supplementary Figure S1.** Components of LHS1 (a), LHS2 (b), LHS3 (c), and LHS4 (d).

Supplement: Figure S1 — Components of LHS1 (A), LHS2 (B), LHS3 (C), and LHS4 (D). (PDF) [file pone.0048022.s001.pdf]

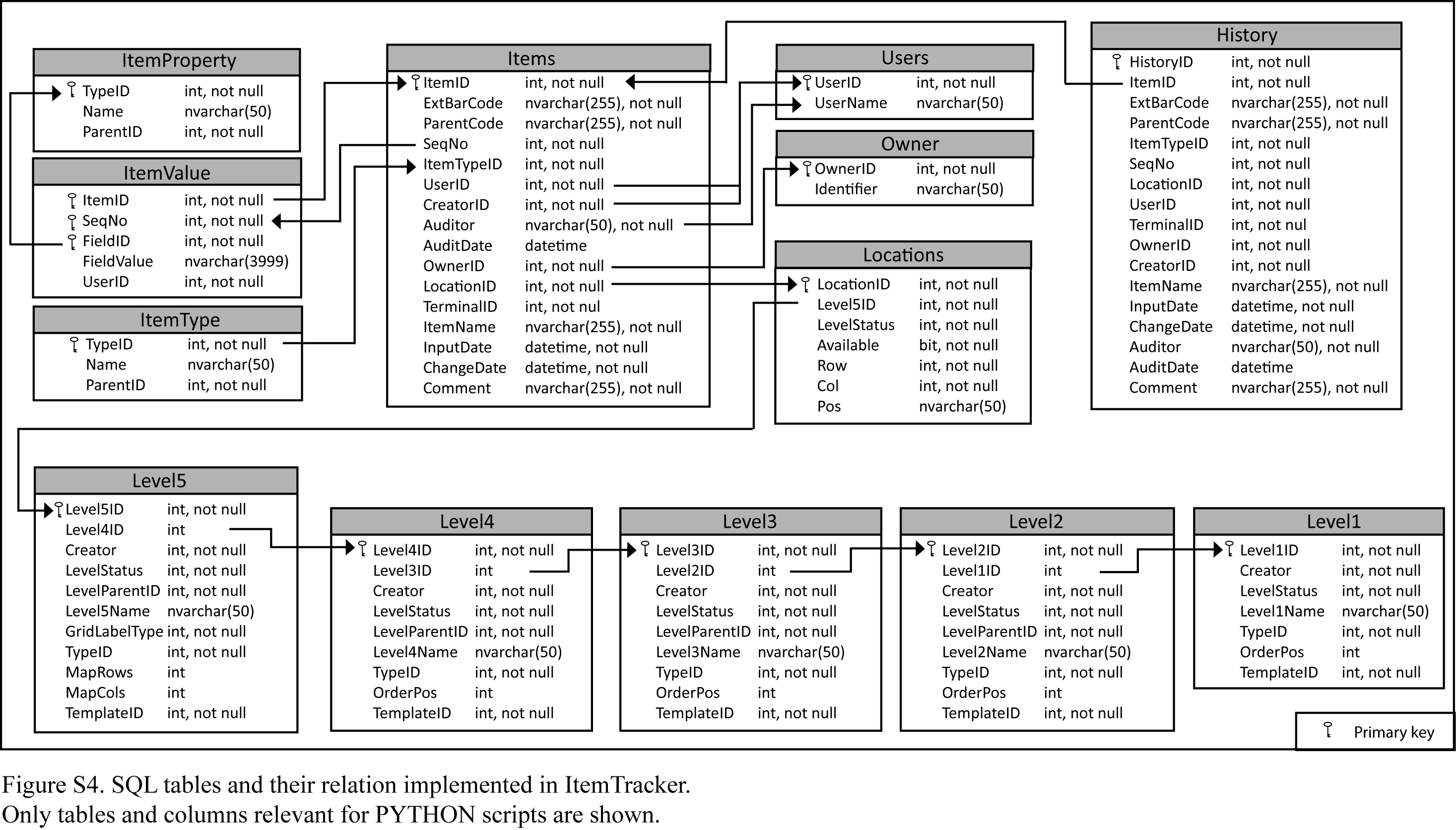

Supplement: Figure S4 — SQL tables and their relation implemented in ItemTracker. Only tables and columns relevant for PYTHON scripts are shown. (TIF) [file pone.0048022.s004.tif]

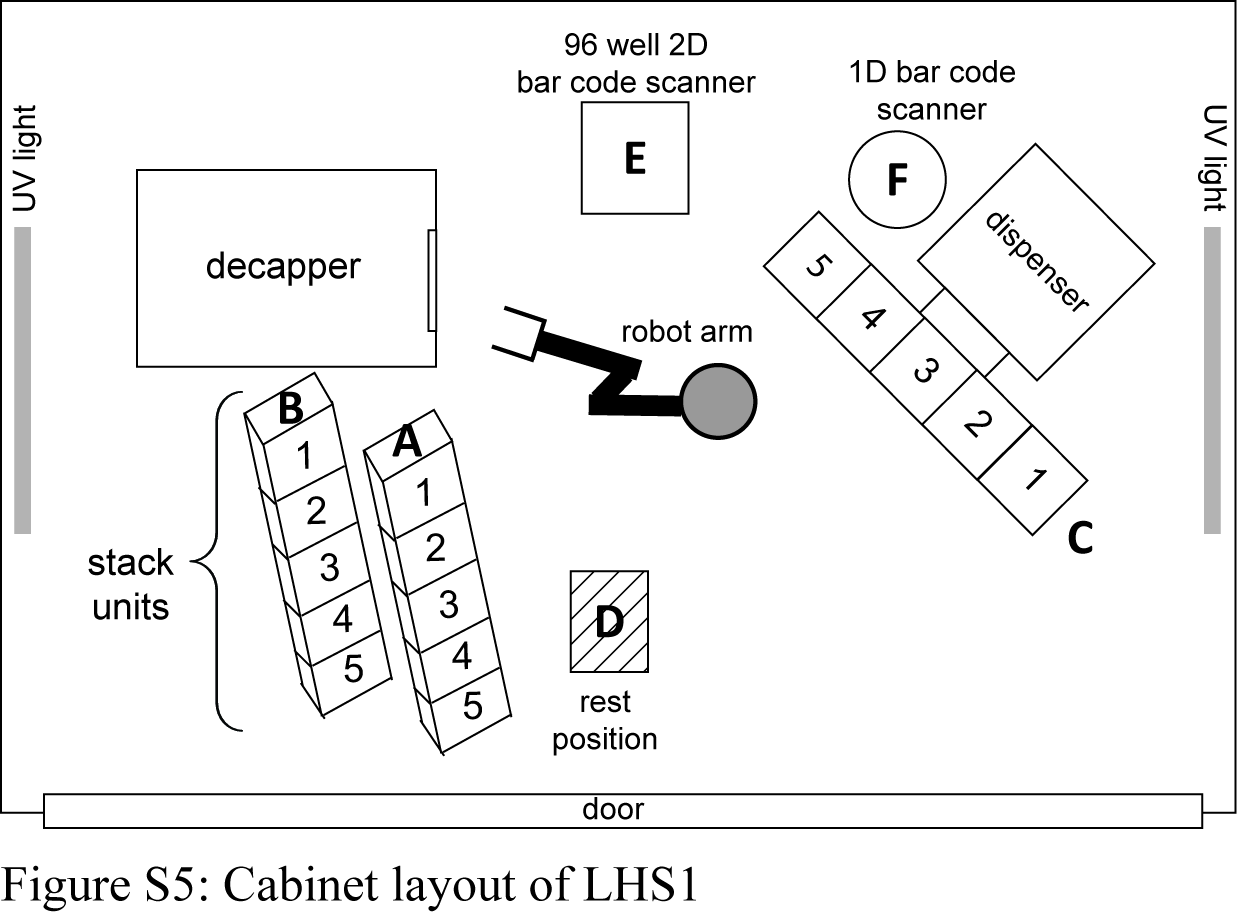

Supplement: Figure S5 — Cabinet layout of LHS1. (TIF) [file pone.0048022.s005.tif]

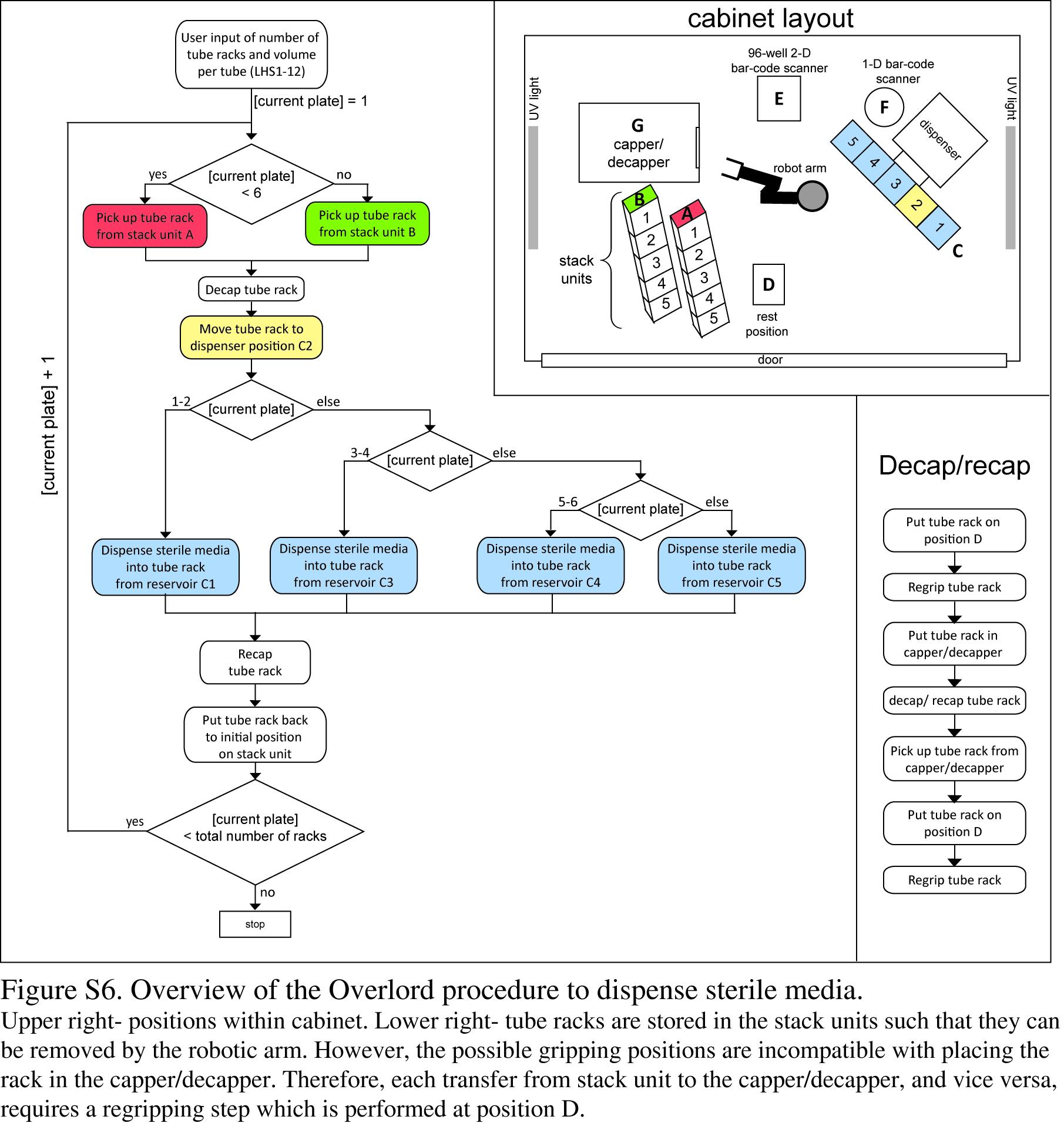

Supplement: Figure S6 — Overview of the Overlord procedure to dispense sterile media. Upper right- positions within cabinet. Lower right- tube racks are stored in the stack units such that they can be removed by the robotic arm. However, the possible gripping positions are incompatible with placing the rack in the capper/decapper. Therefore, each transfer from stack unit to the capper/decapper, and vice versa, requires a regripping step which is performed at position D. (TIF) [file pone.0048022.s006.tif]

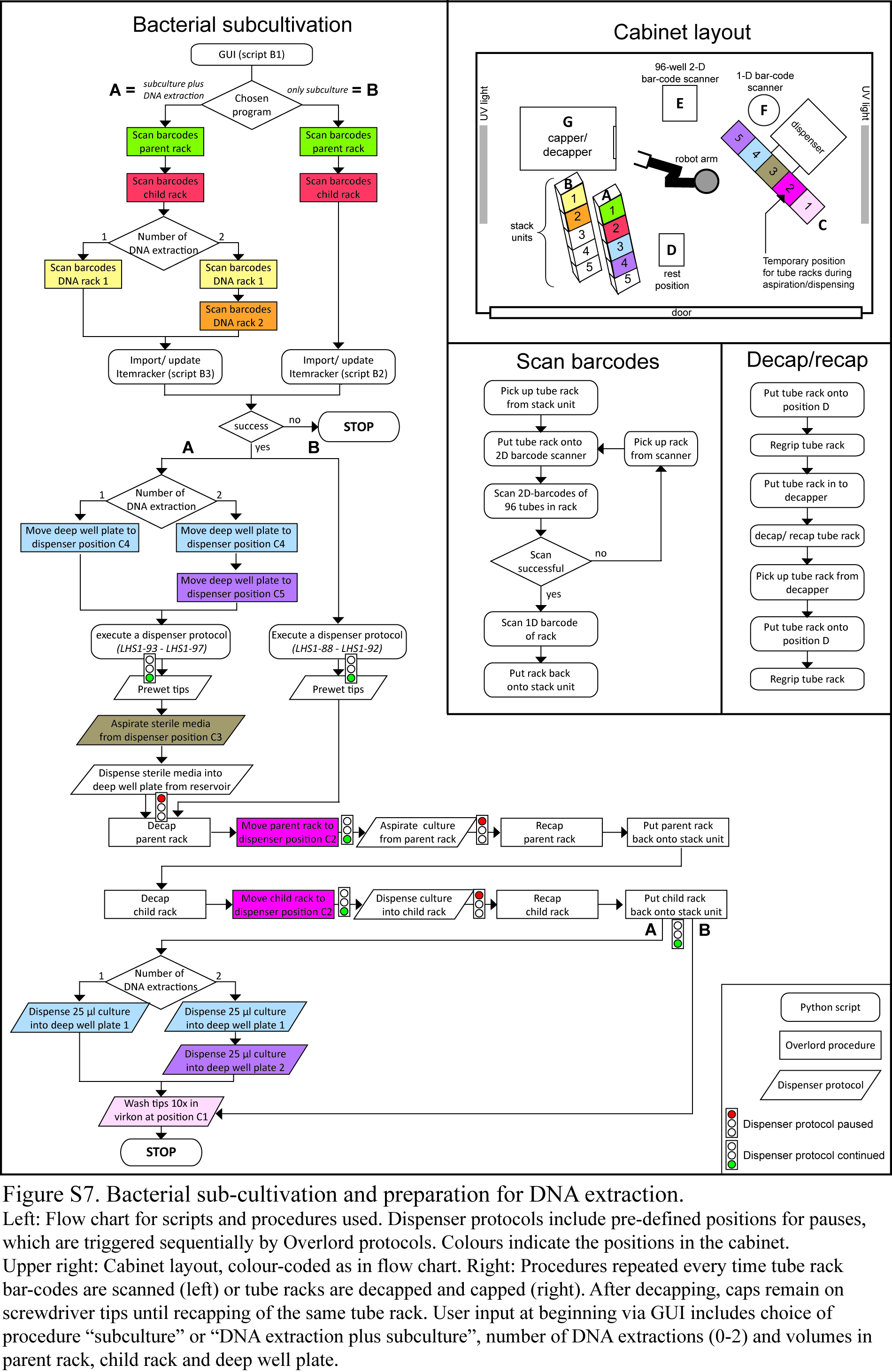

Supplement: Figure S7 — Bacterial sub-cultivation and preparation for DNA extraction. Left: Flow chart for scripts and procedures used. Dispenser protocols include pre-defined positions for pauses, which are triggered sequentially by Overlord protocols. Colors indicate the positions in the cabinet. Upper right: Cabinet layout, color-coded as in flow chart. Right: Procedures repeated every time tube rack barcodes are scanned (left) or tube racks are decapped and capped (right). After decapping, caps remain on screwdriver tips until recapping of the same tube rack. User input at beginning via GUI includes choice of procedure “subculture” or “DNA extraction plus subculture”, number of DNA extractions (0–2) and volumes in parent rack, child rack and deep well plate. (TIF) [file pone.0048022.s007.tif]

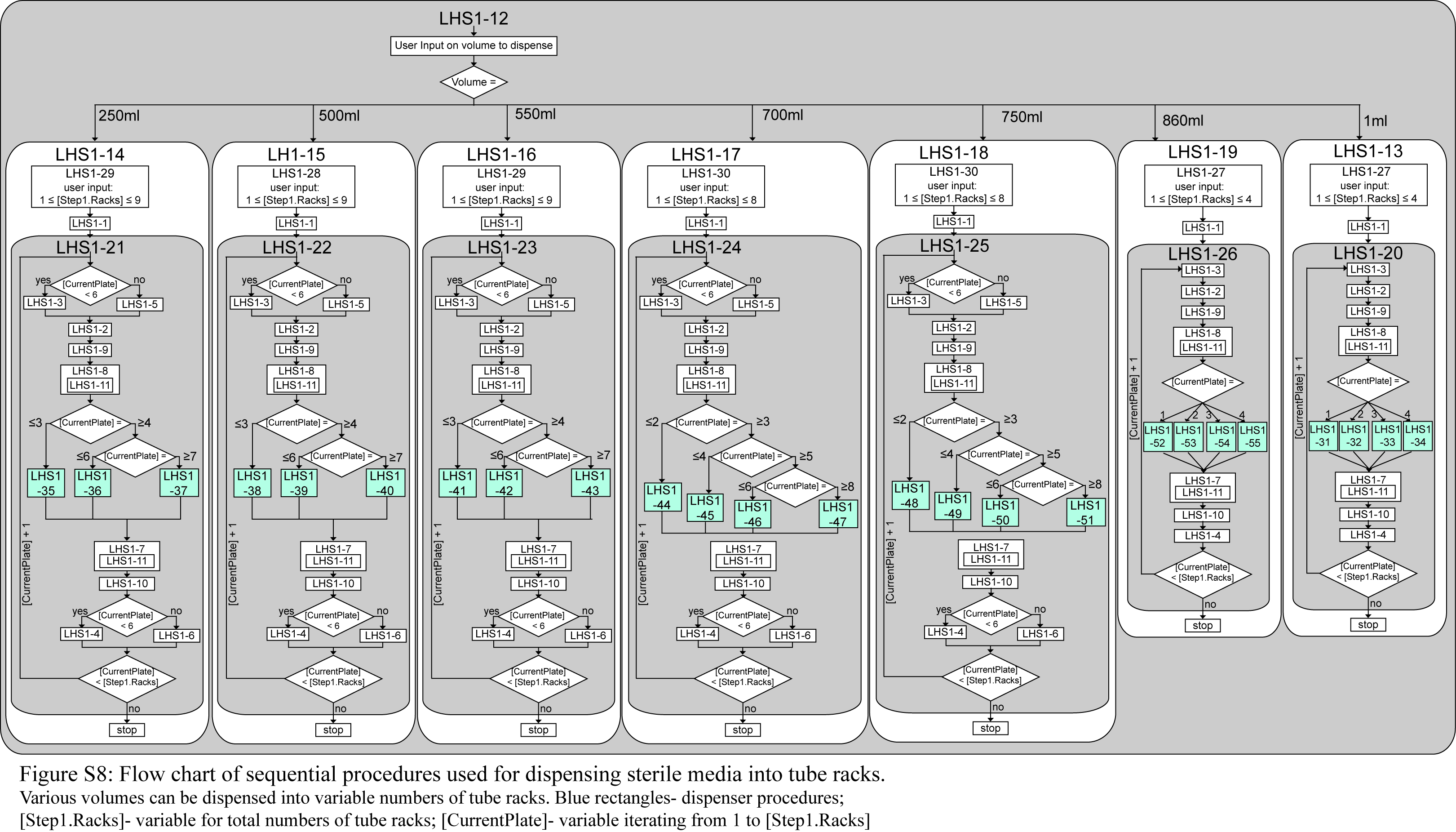

Supplement: Figure S8 — Flow chart of sequential procedures used for dispensing sterile media into tube racks. Various volumes can be dispensed into variable numbers of tube racks. Blue rectangles- dispenser procedures; [Step1.Racks]- variable for total numbers of tube racks; [CurrentPlate]- variable iterating from 1 to [Step1.Racks] (TIF) [file pone.0048022.s008.tif]

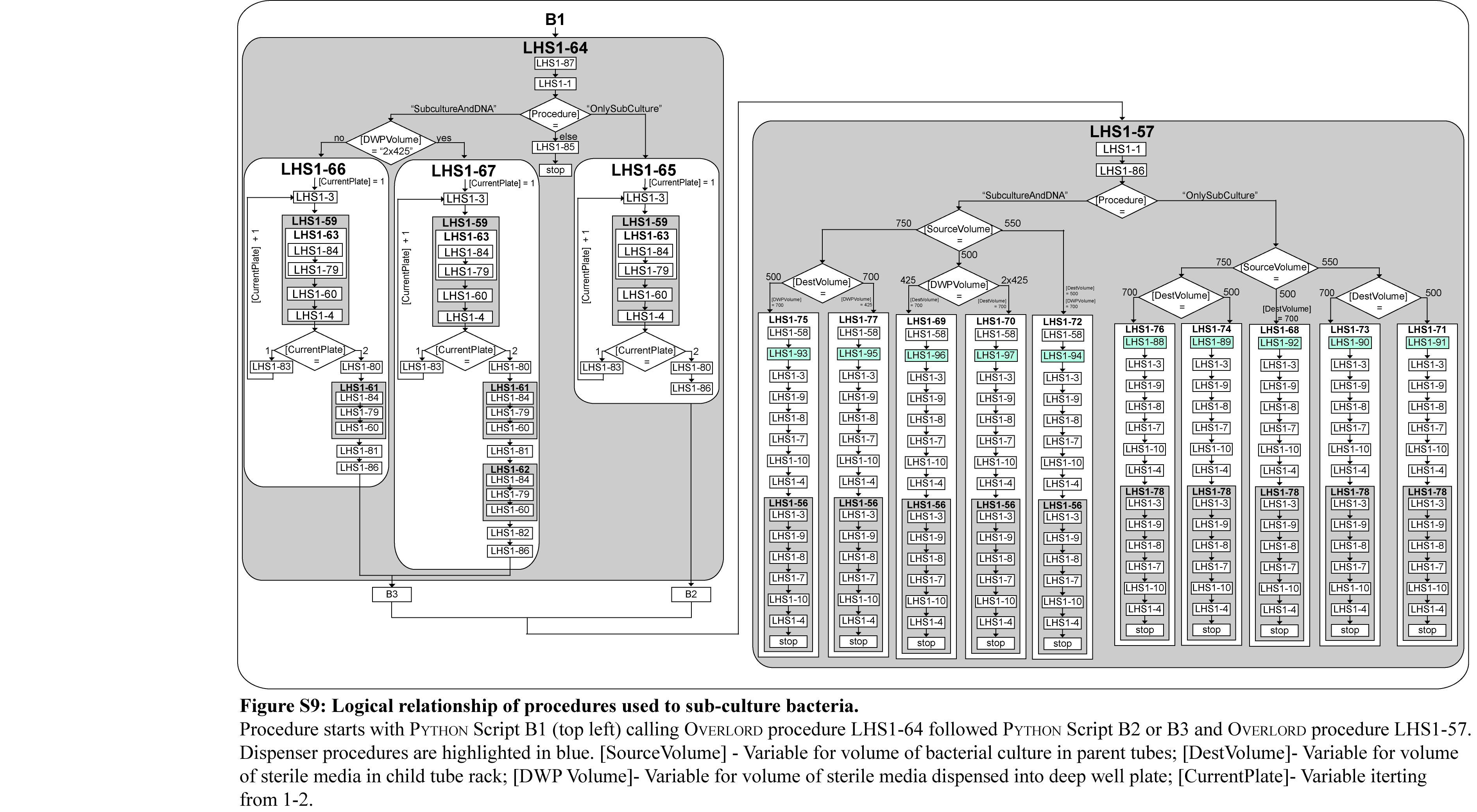

Supplement: Figure S9 — Logical relationship of procedures used to subculture bacteria. Procedure starts with Python Script B1 (top left) calling Overlord procedure LHS1-64 followed Python Script B2 or B3 and Overlord procedure LHS1-57. Dispenser procedures are highlighted in blue. [SourceVolume] - Variable for volume of bacterial culture in parent tubes; [DestVolume]- Variable for volume of sterile media in child tube rack; [DWP Volume]- Variable for volume of sterile media dispensed into deep well plate; [CurrentPlate]- Variable iterting from 1–2. (TIF) [file pone.0048022.s009.tif]
