# Supplementary material for: Transforming Microbial Genotyping: A Robotic Pipeline for Genotyping Bacterial Strains
Source: PLoS One. 2012 Oct 29;7(10):e48022. doi: 10.1371/journal.pone.0048022 (PMC3483277; doi:10.1371/journal.pone.0048022)
Supplement: Figure S2 — Screenshots of the two primary GUI Scripts used in Module A. (A) Script A10 shows the contents of 2D tubes after bar-code scanning. 96 square buttons show the contents of ‘StrainID’ and ‘ItemName’ for each 2D tube. These are color-coded according to five categories, three of which indicate discrepancies between the tubes scanned and data in the SMS. An x symbol in a button indicates that no tube was present at that position. Clicking on one of the 96 square buttons opens a small sub-window with additional information from the SMS for the scanned bar-code and the rack location. Nine other rectangular buttons are included at the top, two of which are color-coded. Clicking on the colored button labelled “Remove/delete items” opens a vertical list of all discrepant tubes. Radio buttons associated with each tube facilitate choosing tubes to be deleted, or whose location should be deleted. “Move items” allows updating tube locations in the SMS with their current locations in this rack. The SMS also stores information on items that are “selected” by Script A6. Such selected items are colored green when “Show selected Items” is clicked. Similarly, “Show status of FrozenStock” distinguishes between the status “Confirmed” (green) and “Contaminated” (red). (B) Script A9 is used during manual microbiology. It creates new items and updaties existing linked items in the SMS. Each button offers the opportunity to compare the results of one or two bar-codes with one or two other bar-codes during various manipulations of bacteria on plates, in shipping containers or 2D tubes during transfer of material, sub-cultivation or DNA extraction. (PDF) [file pone.0048022.s002.pdf]

(a)

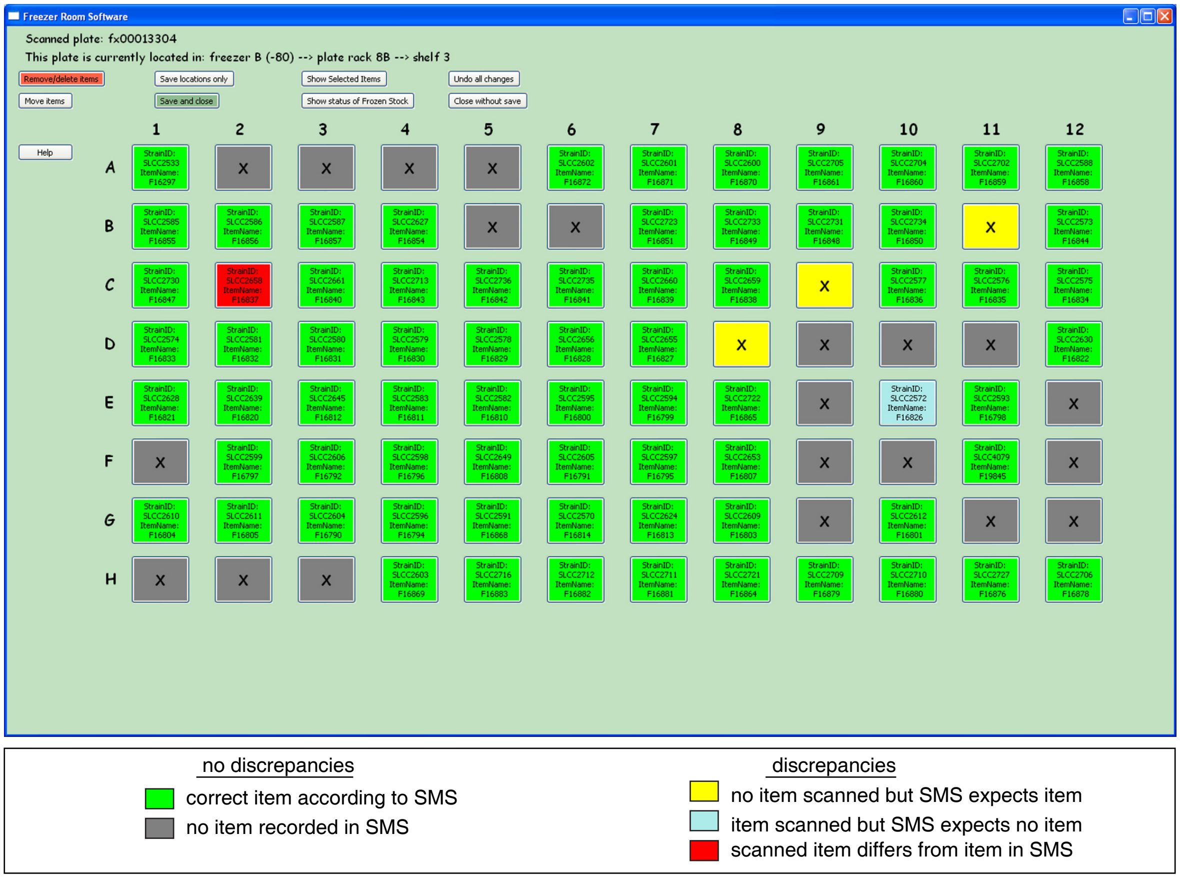

(b)

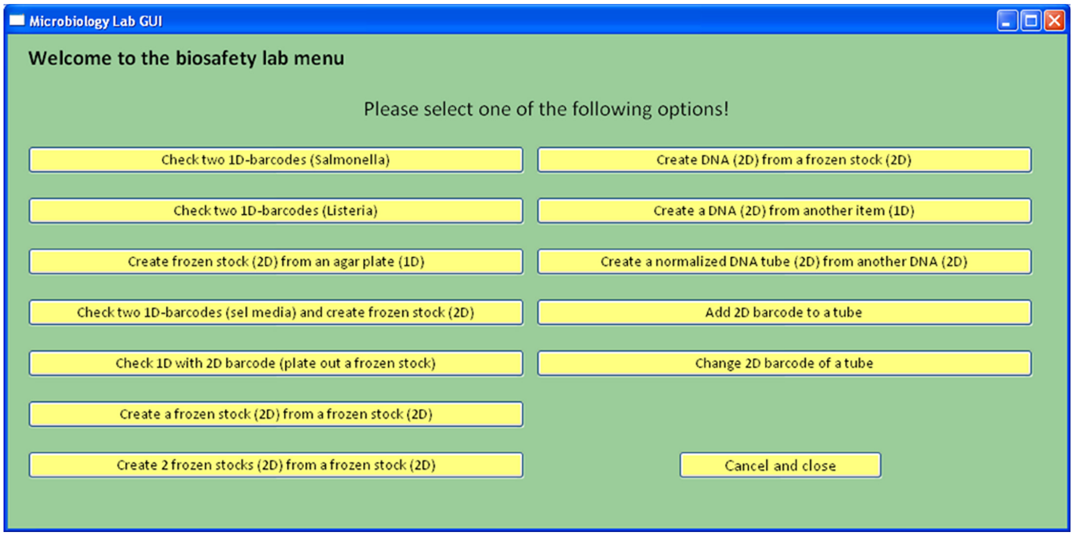

Supplementary Figure S2. Screenshots of the two primary GUI Scripts used in Module A.
a) Script A10 shows the contents of 2D tubes after bar-code scanning. 96 square buttons show the contents of ‘StrainID’ and ‘ItemName’ for each 2D tube. These are colour-coded according to five categories, three of which indicate discrepancies between the tubes scanned and data in the SMS. An x symbol in a button indicates that no tube was present at that position. Clicking on one of the 96 square buttons opens a small sub-window with additional information from the SMS for the scanned bar-code and the rack location. Nine other rectangular buttons are included at the top, two of which are colour-coded. Clicking on the coloured button labelled “Remove/delete items” opens a vertical list of all discrepant tubes. Radio buttons associated with each tube facilitate choosing tubes to be deleted, or whose location should be deleted. “Move items” allows updating tube locations in the SMS with their current locations in this rack. The SMS also stores information on items that are “selected” by Script A6. Such selected items are coloured green when “Show selected Items” is clicked. Similarly, “Show status of FrozenStock” distinguishes between the status “Confirmed” (green) and “Contaminated” (red).
b) Script A9 is used during manual microbiology. It creates new items and updaties existing linked items in the SMS. Each button offers the opportunity to compare the results of one or two bar-codes with one or two other bar-codes during various manipulations of bacteria on plates, in shipping containers or 2D tubes during transfer of material, sub-cultivation or DNA extraction.
