# Supplementary material for: Transforming Microbial Genotyping: A Robotic Pipeline for Genotyping Bacterial Strains
Source: PLoS One. 2012 Oct 29;7(10):e48022. doi: 10.1371/journal.pone.0048022 (PMC3483277; doi:10.1371/journal.pone.0048022)
Supplement: Figure S3 — Screenshot of Scripts that link Bionumerics with the SMS and CherryPicking databases. (A) Script A6 shows the properties of individual stab cultures as well as 2D tubes containing frozen stocks or DNAs within the SMS. This window initially shows bacterial strains that were selected in Bionumerics prior to running the script. The contents of the window can be changed by clicking on buttons at the top left: “Get All Selected” retrieves items in the Bionumerics database which were previously selected in the SMS by the user; “CherryPicking” displays all DNAs of strains in the Bionumerics database which are listed in the CherryPicking database with the status “Repeat”. Left panel: Tree-like representation of the parent-child hierarchy in the SMS, including selected fixed properties, such as ‘viability F’ or ‘DNA concentration’. Items without a location in the SMS are labelled in red, while items that were previously selected are marked in green. Ticked items can be selected/deselected through the Update button at the lower left. Right Panel: Additional freely selectable information from Bionumerics and the ItemTracker SMS for the highlighted item in the left panel. The sources of this information are chosen with the buttons ‘ItemTracker’ and ‘Bionumerics’ at the lower right. Bottom panel: Log of changes in selection status. (B) Script D10 script automatically attempts to complete all unfinished contigs in Bionumerics. For contigs containing two or more traces, it attempts to assign both trimming sites and checks whether each nucleotide between those trimming sites is covered by at least two traces, and that there are no discrepancies between traces. Contigs that meet these criteria are saved as finished in Bionumerics. Otherwise an assembly window is opened (not shown) as well as the control panel shown here, to give the user an opportunity to manually resolve problems or choose traces which need to be repeated. For traces that need to be repeated, the user can specify w [file pone.0048022.s003.pdf]

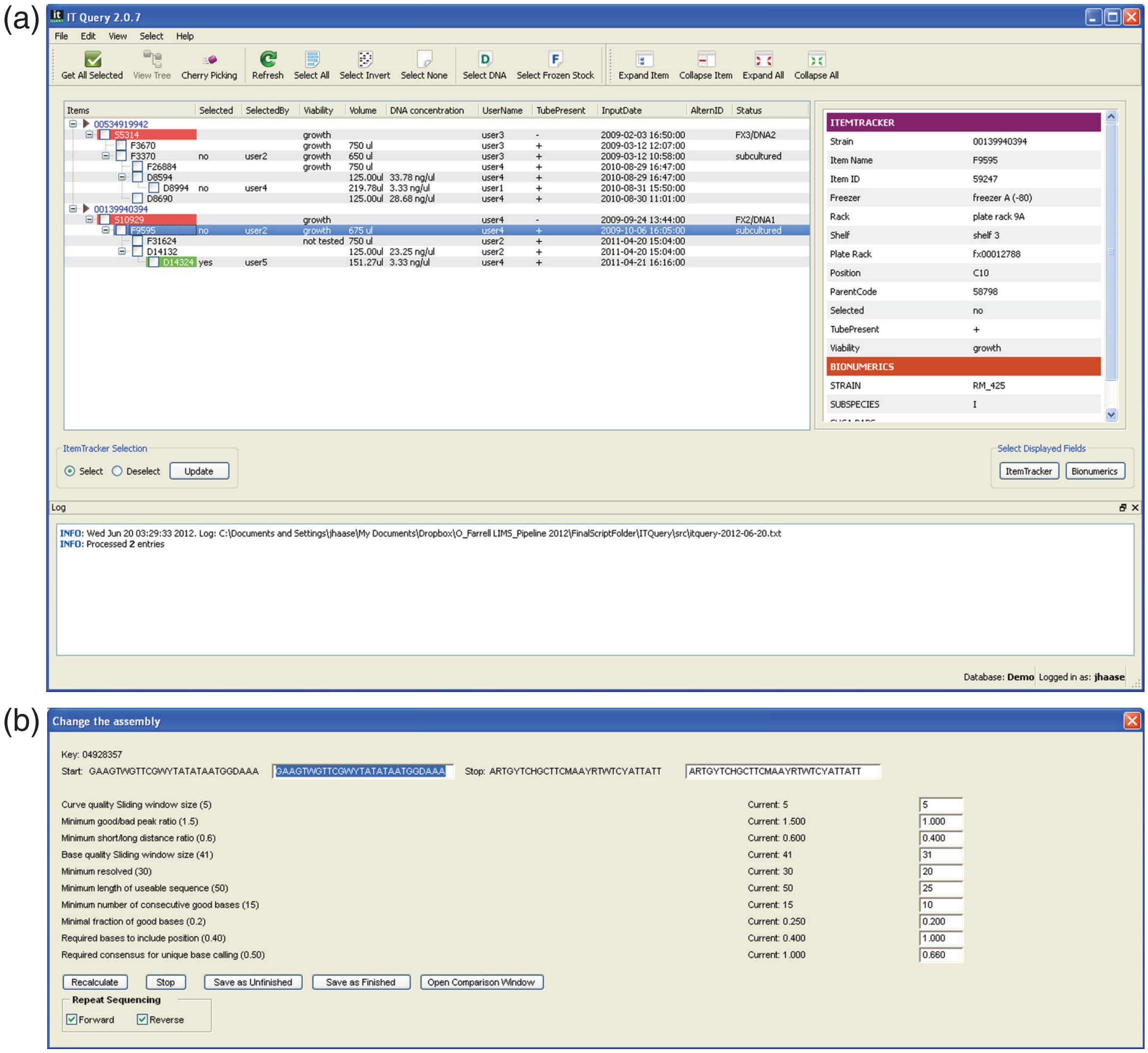

Fig. S3. Screenshot of Scripts that link BIONUMERICS with the SMS and CHERRYPICKING databases.
a) Script A6 shows the properties of individual stab cultures as well as 2D tubes containing frozen stocks or DNAs within the SMS. This window initially shows bacterial strains that were selected in BIONUMERICS prior to running the script. The contents of the window can be changed by clicking on buttons at the top left: "Get All Selected" retrieves items in the BIONUMERICS database which were previously selected in the SMS by the user; "CherryPicking" displays all DNAs of strains in the BIONUMERICS database which are listed in the CHERRYPICKING database with the status "Repeat". Left panel: Tree-like representation of the parent-child hierarchy in the SMS, including selected fixed properties, such as 'viability F' or 'DNA concentration'. Items without a location in the SMS are labelled in red, while items that were previously selected are marked in green. Ticked items can be selected/deselected through the Update button at the lower left. Right Panel: Additional freely selectable information from BIONUMERICS and the ITEMTRACKER SMS for the highlighted item in the left panel. The sources of this information are chosen with the buttons 'ItemTracker' and 'Bionumerics' at the lower right. Bottom panel: Log of changes in selection status.
b) Script D10 script automatically attempts to complete all unfinished contigs in BIONUMERICS. For contigs containing two or more traces, it attempts to assign both trimming sites and checks whether each nucleotide between those trimming sites is covered by at least two traces, and that there are no discrepancies between traces. Contigs that meet these criteria are saved as finished in BIONUMERICS. Otherwise an assembly window is opened (not shown) as well as the control panel shown here, to give the user an opportunity to manually resolve problems or choose traces which need to be repeated. For traces that need to be repeated, the user can specify whether the forward and/or reverse traces need repetition (lower left), and this information is stored in the CHERRYPICKING database.
