# Supplementary material for: Transforming Microbial Genotyping: A Robotic Pipeline for Genotyping Bacterial Strains
Source: PLoS One. 2012 Oct 29;7(10):e48022. doi: 10.1371/journal.pone.0048022 (PMC3483277; doi:10.1371/journal.pone.0048022)
Supplement: Table S1 — Columnnames in table “traces” of cherrypicking database. (DOCX) [file pone.0048022.s010.docx]

Table S1. Column names in the “traces” table of the cherrypicking database.

| Column name | Description |
| --- | --- |
| id^1^ | Sequential number |
| key | Strain identifier |
| gene | Name of gene to be repeated |
| orientation | Sequence direction to be repeated (choice: *forward*/*reverse*) |
| database | Name of Bionumerics database where strain information and sequences are stored |
| username | User who evaluated trace |
| date | Time stamp of entry |
| status | Status of sequence (choice: *Repeat*/*Picked*/*Finished*) |

^1^Primary key
