# Supplementary material for: Transforming Microbial Genotyping: A Robotic Pipeline for Genotyping Bacterial Strains
Source: PLoS One. 2012 Oct 29;7(10):e48022. doi: 10.1371/journal.pone.0048022 (PMC3483277; doi:10.1371/journal.pone.0048022)
Supplement: Table S2 — Scripts developed for the pipeline. (DOCX) [file pone.0048022.s011.docx]

Table S2. Scripts developed for the pipeline.

| Script identifier | Name of Script | Description |
| --- | --- | --- |
| **A- Cultivation and Storage** | |  |
| A1 | ItemTrackerModule.py | Global functions to retrieve and alter data in SMS. Used by all scripts to connect to SMS. |
| Enter Strain information | |  |
| A2 | ImportStrainIntoItemTracker.py | Creates ‘Bacteria’ item with Strain designations in SMS and additionally one ‘Salmonella’, ‘Listeria’ or ‘Ecoli’ item. User selects species and type of culture via a GUI. Output file contains strain designations from input file and the corresponding unique key from SMS (ItemID). |
| A3 | BNInput.py | Checks for potential spelling mistakes or inconsistent field values in the Excel file containing strain information. Prepares an input file for import into Microsoft Access. |
| A4 | MergeStrainIDWithItemID.py | Collates information on strains from output file of script A3 with output file of script A2. |
| A5 | import_access.py | Transfers strain information from Microsoft Access into Bionumerics. |
| A6 | ITQuery.py | GUI which is run within Bionumerics to visualize all derived items of a strain in SMS which the user can either select or unselect (Fig. S3a). |
| Manual lab work | |  |
| A7 | label_print.py | Prints labels with ItemName from SMS as text and bar-code plus the StrainID as text based on information from a text file (either direct export from SMS or output file from A8). |
| A8 | ProduceLabelFile.py | Creates input file for script A7 to print labels based on scanned bar-codes (ItemNames). |
| A9 | MicrobiologyLabGUI.py | GUI that accompanies all manual lab work in a sterile cabinet, which checks bar-codes for identities and updates the SMS (Fig. S2b). |
| A10 | FreezerRoomSoftware.py | GUI to provide information on the contents of a 2D tube rack and allow updating of SMS (Fig. S2a). |
| A11 | ListFiles.py | Gets a list of files in a folder with a file name pattern. Used by script A9 and A10. |
| Listeria manual pipeline | |  |
| A12 | ConfirmLiseria.py | Records positive result from selective agar for ‘Listeria’ item in SMS. |
| A13 | NotListeria.py | Records negative result from selective agar for ‘Listeria’ item in SMS. |
| A14 | GetStatusOfStrain.py | Retrieves information from SMS about items via the scanned bar-code, e.g. ‘viability L’ or ‘status L’. |
| A15 | StrainsDidNotGrow.py | Records negative result about growth of ‘Listeria’ SMS. Chooses alternative sister tube of a ‘Listeria’ for growth. |
| A16 | UpdateAcessAndITAndBN6.BNS | Transfers strain information into Bionumerics from Microsoft Access where it has been entered manually from written records via an entry form. Only information of viable strains is transferred. |
| **B- DNA Extraction Normalisation** | |  |
| B1 | SubcultureBacteria.py | GUI to choose procedure to run LHS1 wherein sub-culturing bacteria and culture transferring into deep well plate is performed. Script calls B2 and B3 for updating SMS. |
| B2 | FluidXOnlyTubeRack.py | Creates ‘Frozen Stock’ items in SMS based on information in a text file created by LHS1. ‘Volume F’ and ‘status F’ of parent ‘Frozen Stock’ is updated (called by Script B1). |
| B3 | FluidXTubeRackAndDWP.py | Creates ‘Frozen Stock’ and ‘DNA’ items in SMS based on information in a text file created by LHS1. ‘Volume F’ and ‘status F’ of parent ‘Frozen Stock’ are updated (called by Script B1). |
| B4 | ItemTrackerFunctions.py | Global functions used by scripts in module C, D and E to create ‘DNA’, ‘PCR product’ and ‘Sequencing reaction’ in SMS. 384/96-well plates (table Level5) and Locations (table Locations) are also created in SMS. |
| B5 | SELECTOR.py | A master GUI where the user selects which procedure they wish to run on LHS4. |
| B6 | WinPrepControl.py | This script contains a series of important scripts which are used to control LHS4 procedures. |
| B7 | user_input.py | Takes user input for sample number. |
| B8 | ConnectToXtr96_DestPlate.py | Driver for 2-D bar-code scanning of empty tubes in destination plate and data handling. |
| B9 | ConnectToXtr96.py | Driver for 2-D bar-code scanning of source DNA plate and data handling. |
| B10 | linefitter.py | Script for linear regression on the standard curve data generated during LHS4 normalisation. |
| B11 | VictorDNACalc.py | Driver which activates fluorimeter reads, calculates DNA concentrations and creates the pipetting layout for LHS4 normalisation. |
| B12 | MeasureDNAConc_PrepareWorkingSol.py | Script to interact with the SMS during LHS4 normalisation for creating normalized DNA items and updating volumes and DNA concentrations. |
| B13 | NOT_NORMALISED.py | Script indicates to the user which, if any, DNAs were not normalised to target concentration. |
| B14 | PrepareWorkingSolution_BOF.py | Script to assist the creation of new normalised ‘DNAs’ from known concentrations of ‘DNA’ in SMS by calculating the correct pipetting pattern. |
| B15 | Standard_Norm_8_03.MPT | Standard normalisation procedure. |
| B16 | Normalisation_Itemtracker_v1.0.MPT | Normalisation based on known DNA concentrations from SMS. |
| B17 | Macherey-Nagel 20110512.CAS4 | DNA extraction protocol on LHS2. |
| **C- Semi-automated PCR and Cleanup** | |  |
| C1 | FreezerRoomTypeScan.py | Driver for 2-D bar-code scanner used in MLST. |
| C2 | mlst_gui.py | This script handles interactions with the SMS during PCR and Sequencing reaction setup. |
| C3 | GetKeysFromBN.py | Script for checking ‘ItemTracker IDs’ in Bionumerics and changing status of cherrypicked DNAs in CherryPicking to ‘picked’ |
| C4 | LocusWSSelectFromDB.py | This script interacts with the SMS and provides a GUI for PCR setup on LHS4 where the user selects what genes and primer working solutions are being used. It also shows the user where to place primer mix tubes on the deck. |
| C5 | CP_PCR_01_01.MPT | Semi-automated PCR setup procedure on LHS4. |
| Manage Oligonucleotides | |  |
| C6 | ImportOligos.py | Imports new oligonucleotides into SMS based on information in an Excel file. Creates output file which is used directly for ordering oligonucleotides from supplier. |
| C7 | CreateWS_label_print.py | Printing bar-codes from output file of C8. |
| C8 | CreateWS.py | GUI which allows the user to create new primer ‘Working stock’ items in SMS which are a combination of oligonucleotides. |
| **D- Semi-automated Sequencing** | |  |
| D1 | DIL_mlst.py | Creates new diluted ‘PCR products’ in SMS using plate bar-code information when PCRs are being diluted into new plates with LHS4. |
| D2 | SEQ_mlst.py | SMS interaction, data handling during creation of sequencing products, files created for downstream sequencing (names for trace files used by sequencing facility). |
| D3 | seqmmx_mlst.py | Retrieves information from SMS on 384-well plates containing dried PCR template. |
| D4 | SequencingWSSelectFromDB.py | GUI which shows user where to place primer tubes during sequencing step. |
| D5 | 384DIL_SETUP_02_01.MPT | Semi-automated PCR dilution procedure on LHS4. |
| D6 | 384SEQ_TEMPLATE_03_02.MPT | Semi-automated PCR template dispense for sequencing reaction set up on LHS4. |
| D7 | 384SEQ_MMX_ADDITION_01_01.MPT | Semi-automated sequencing mastermix dispense to plates with dried template on LHS4. |
| D8 | InsertNewTraces_bn6.BNS | Imports trace files into Bionumerics. (Uses generic functions in Script D9). |
| D9 | Funcsinsert_bn6.bns | Functions to insert new traces which are used by Script D8. |
| D10 | Iterate3.bns | Iterates over selected entries and assembles and trims unapproved sequences in Bionumerics. Script will approve assemblies that fulfil criterion of full coverage of the allele with at least two traces and agreement between traces. Alternatively, assembly window opens and allows manual editing of the sequences. If user decides to repeat a sequence, the script will enter identifiers for repeats into CherryPicking which is updated accordingly if repeated sequences are approved. |
| D11 | FunctionsIterate3_bn6 | Functions used by Script D10 to assess quality of traces and enter failed sequences into Cherrypicking. |
| D12 | DeleteFolder.py | Used by Script D8 to delete a folder. |
| **E- Robotic Sequencing** | |  |
| E1 | Cherry_Select_Species.py | GUI for SMS interaction used in Cherrypicking PCR setup. |
| E2 | cherrypickingjanusfiles.py | Identifies sequences to be repeated for scanned DNAs for user-selected genes, creates optimal layout (DNA, gene and orientation) and creates files for LHS4 pipetting procedure, tells user where to place primer mix tubes, updates status for appropriate entries in cherrypicking. |
| E3 | CherryPicker_DIL_mlst.py | Script interacts with SMS and creates new diluted ‘PCR products’ in SMS during Cherrypicking |
| E4 | CherryPicking_seq.py | Script for data handling and creation of ‘Sequencing products’, creating files for sequencing facility during Cherrypicking. |
| E5 | CherryPicking_UNIVERSAL_Seq.py | Script for data handling and creation of ‘Sequencing products’, creating files for sequencing facility during Cherrypicking when universal Sequencing primers are being used. |
| E6 | CHERRYPICKING_SequencingWSSelectFromDB.py | GUI for selecting ‘Working Stocks’ for sequencing during in cherrypicking. |
| E7 | CP_PCR_01_01.MPT | Robotic sequencing PCR setup LHS4 procedure. |
| E8 | CP_DIL_WITHOUT_RED_01_01.MPT | Robotic sequencing PCR dilution LHS4 procedure. |
| E9 | CP_SEQ_WITHOUT_RED_01_01.MPT | Standard robotic sequencing reaction setup LHS4 procedure. |
| E10 | CP_UNIV_SEQ_WITHOUT_RED_01_01.MPT | Robotic sequencing reaction setup LHS4 procedure where universal sequencing primers are being used. |
| E11 | CherryPicker_CreateRack.py | Script to calculate optimal combinations of DNAs to fill 384-well plates for repeats for user specified species and genes. Locates DNAs for suggested plates. Used again in downstream Cherrypicking. |

SMS - sample management system
