# Supplementary material for: Transforming Microbial Genotyping: A Robotic Pipeline for Genotyping Bacterial Strains
Source: PLoS One. 2012 Oct 29;7(10):e48022. doi: 10.1371/journal.pone.0048022 (PMC3483277; doi:10.1371/journal.pone.0048022)
Supplement: Table S3 — Common, predefined properties that apply to all items. (DOCX) [file pone.0048022.s012.docx]

Table S3. Common, predefined properties that apply to all items.

| Property (SQL table: Items) | Description |
| --- | --- |
| ItemID | Unique identifier |
| ExtBar code | 2D bar code |
| ParentCode | ItemID of parent Item |
| SeqNo | Identifier for historical state including current state |
| ItemTypeID | Identifier for the type of Item |
| UserID | Identifier for user who last changed the Item |
| CreatorID | User who created this Item |
| Auditor | User who last audited this Item |
| AuditDate | Date of last audit |
| OwnerID | Identifier of Owner (“UCC” in our setup) |
| LocationID | Identifier for the location of this Item |
| TerminalID | PC with which the Item was created |
| ItemName | Identifier for type of Item plus sequential number which is used for 1D bar codes |
| InputDate | Date when item was created |
| ChangeDate | Date when item was last changed |
| Comment | Any comments |
