# Supplementary material for: Transforming Microbial Genotyping: A Robotic Pipeline for Genotyping Bacterial Strains
Source: PLoS One. 2012 Oct 29;7(10):e48022. doi: 10.1371/journal.pone.0048022 (PMC3483277; doi:10.1371/journal.pone.0048022)
Supplement: Table S4 — ItemTypes and ItemProperties defined by administrator. (DOCX) [file pone.0048022.s013.docx]

Table S4. ItemTypes and ItemProperties defined by administrator.

| ItemType | ItemProperty* | Data type | Description |
| --- | --- | --- | --- |
| All types | Selected | Yes/no | Item can be selected or unselected |
|  | Selected by | Text | User who selected the Item |
| Bacteria | BionumericsKey | Text | Unique identifier in sequencing analysis database (usually identical to StrainID) |
|  | StrainID† | Text | Unique strain identifier in ItemTracker which is inherited by all derived children |
|  | Original_ID | Text | Original strain identifier |
|  | Altern_ID | Text | Alternative strain identifier |
|  | Species | Text | Choice: ‘E. coli’, ‘S. enterica’, ‘Listeria’, ‘Unknown’ |
| Salmonella | Salm_ID | Number | Sequential number to build ItemName (letter code ‘S’ plus sequential number) |
|  | Status S | Text | Status of tube in the pipeline (e.g. ‘growing in plate’) |
|  | Type of culture S | Text | Choice: ‘stab’, ‘lyophil’, ‘slant’ |
|  | Viability S | Text | Choice: ‘growth’, ‘no growth’, ‘contaminated’ |
| Listeria | Lis_ID | Number | Sequential number to build ItemName (letter code ‘L’ plus sequential number) |
|  | Status L | Text | Status of tube in the pipeline (e.g. ‘growing in plate’) |
|  | Type of culture L | Text | Choice: ‘stab’, ‘lyophil’, ‘slant’ |
|  | Viability L | Text | Choice: ‘growth’, ‘no growth’, ‘contaminated’ |
| Ecoli | Ecoli_ID | Number | Sequential number to build ItemName (letter code ‘E’ plus sequential number) |
|  | Status E | Text | Status of tube in the pipeline (e.g. ‘growing in plate’) |
|  | Type of culture E | Text | Choice: ‘stab’, ‘lyophil’, ‘slant’ |
|  | Viability E | Text | Choice: ‘growth’, ‘no growth’, ‘contaminated’ |
| Frozen Stock | Stock_ID | Number | Sequential number to build ItemName (letter code ‘F’ plus sequential number) |
|  | Freezing medium | Text | Choice: ‘Freezing buffer’, ‘20% glycerol’, ‘10% skim milk’ |
|  | Status F | Text | Status of tube in the pipeline (e.g. ‘subcultured’) |
|  | Viability F | Text | Choice: ‘growth’, ’no growth’, ‘contaminated’ |
|  | Volume in ul F | Text | Total volume of the frozen culture in µl |
| DNA | DNA_ID | Number | Sequential number to build ItemName (letter code ‘D’ plus sequential number) |
|  | Concentration (ng/ul) | Text | Concentration of the DNA in ng per µl |
|  | Adjust conc | Yes/no | Yes if DNA has been adjusted to a defined concentration |
|  | Date | Date | Date of DNA extraction |
|  | Status D | Text | Status of tube in the pipeline (e.g. ‘subcultured’) |
|  | Volume in ul | Text | Total volume of DNA in µl |
| PCR product | PCR_ID | Number | Sequential number to build ItemName (letter code ‘P’ plus sequential number) |
|  | Primers P | Text | ItemName of ‘working stock’ used for PCR reaction |
|  | Gene P | Text | Amplified gene |
|  | Numbers of cycles P | Text | Number of cycles used in PCR program |
|  | Status P | Text | Status of tube in the pipeline (e.g. ‘PCR prepared’) |
|  | Volume in ul P | Text | Total reaction volume |
| Continued on next page. | |  |  |
| Continued from previous page. | |  |  |
| ItemType | Property* | Data type | Description |
|  | Well format P | Text | Microwell plate format (e.g. ‘384’) |
| Sequencing reaction | Seq_ID | Number | Sequential number to build ItemName (letter code ‘Seq’ plus sequential number) |
|  | Status Seq | Text | Status of tube in the pipeline (e.g. ‘Sent off’) |
|  | Volume in ul Seq | Text | Total reaction volume |
|  | Gene Seq | Text | Sequenced gene |
|  | Seq primer | Text | ItemName of Working Stock used for sequencing reaction |
|  | Direction Seq | Text | Direction sequenced (Choice: ‘forward’, ‘reverse’) |
| Oligonucleotide | Oligo_ID | Number | Sequential number to build ItemName (letter code ‘OC’ plus sequential number) |
|  | Oligoname | Text | Trivial name of primer |
|  | Organism | Text | Organism the oligonucleotide was designed for (e.g. ‘S.enterica’) |
|  | Partner oligo | Text | ItemName of oligonucleotide used together with this Item for PCR |
|  | Product length | Text | Length of PCR product (with partner oligo) |
|  | Project | Text | General description of project category (e.g. ‘MLST’) |
|  | Purpose | Text | Choice: ‘PCR’, ‘PCR/Sequencing’, ‘Sequencing’, ‘SNP’, ‘ASPE’, ‘CRISPR’, ‘CRISPOL’ |
|  | Sequence (5-->3) | Text | Nucleotide sequence |
|  | Supplier | Text | Supplier and manufacturer |
|  | Melting temperature | Text | Melting temperature |
|  | Gene fragment/SNP | Text | Gene or single nucleotide polymorphism the oligonucleotide is designed for |
|  | Direction | Text | Direction of oligonucleotide (Choice: ‘forward’, ‘reverse’) |
|  | Antitag region | Text | Code of tag region attached to oligonucleotide |
|  | Date of order | Text | Date when Oligonucleotide was ordered (usually the same as Items.InputDate) |
| Working stock | Working Stock_ID | Number | Sequential number to build ItemName (letter code ‘WS’ plus sequential number) |
|  | Conc in uM WS | Text | Concentration of oligonucleotides in mixture |
|  | Gene WS | Text | Gene which the mixture is intended to amplify/sequence |
|  | Primers WS | Text | List of ItemNames of oligonucleotides included in this mixture |
|  | Species WS | Text | Bacterial species for which this ‘Working stock’ was designed |
|  | Volume in ul WS | Text | Total volume of mixture in µl |

*ItemProperty.Name🡪ItemValue.FieldValue; †Trigger (see Field rules) was implemented to ensure that ‘StrainID’ is inherited. StrainID must be configured as unique.
