# Supplementary material for: Transforming Microbial Genotyping: A Robotic Pipeline for Genotyping Bacterial Strains
Source: PLoS One. 2012 Oct 29;7(10):e48022. doi: 10.1371/journal.pone.0048022 (PMC3483277; doi:10.1371/journal.pone.0048022)
Supplement: Table S5 — Purpose of Field rules that were applied to ItemProperties. (DOCX) [file pone.0048022.s014.docx]

Table S5. Purpose of Field rules that were applied to ItemProperties.

| ItemType | Rule name | Purpose |
| --- | --- | --- |
| Bacteria | Set B Code | ItemName for all Bacteria is ‘Bacteria’ |
|  | StrainID error | StrainID cannot be null |
| Salmonella | Hide Salm_ID | Sequential number hidden in ItemTracker interface |
|  | Set S Code | ItemName is ‘S’ + number |
|  | Check ParentCode | ParentCode must exist |
|  | Hide Original_ID | OriginalID hidden in ItemTracker interface |
|  | Hide Altern_ID | Altern_ID hidden in ItemTracker interface |
|  | Hide Strain_ID | StrainID hidden in ItemTracker interface |
|  | Hide species | Species hidden in ItemTracker interface |
| Listeria | Hide Lis_ID | Sequential number hidden in ItemTracker interface |
|  | Set L Code | ItemName is ‘L’ + number |
|  | Check ParentCode | ParentCode must exist |
|  | Hide Original_ID | OriginalID hidden in ItemTracker interface |
|  | Hide Altern_ID | Altern_ID hidden in ItemTracker interface |
|  | Hide Strain_ID | StrainID hidden in ItemTracker interface |
|  | Hide species | Species hidden in ItemTracker interface |
| Ecoli | Hide Ecoli_ID | Sequential number hidden in ItemTracker interface |
|  | Set E Code | ItemName is ‘E’ + number |
|  | Check ParentCode | ParentCode must exist |
|  | Hide Original_ID | OriginalID hidden in ItemTracker interface |
|  | Hide Altern_ID | Altern_ID hidden in ItemTracker interface |
|  | Hide Strain_ID | StrainID hidden in ItemTracker interface |
|  | Hide species | Species hidden in ItemTracker interface |
| Frozen Stock | Hide Stock_ID | Sequential number hidden in ItemTracker interface |
|  | Set F Code | ItemName is ‘F’ + number |
|  | Check ParentCode | ParentCode must exist |
|  | Hide Strain_ID | StrainID hidden in ItemTracker interface |
| DNA | Hide DNA_ID | Sequential number hidden in ItemTracker interface |
|  | Set D Code | ItemName is ‘D’ + number |
|  | Check ParentCode | ParentCode must exist |
|  | Hide Strain_ID | StrainID hidden in ItemTracker interface |
| PCR product | Hide PCR_ID | Sequential number hidden |
|  | Set P Code | ItemName is ‘P’ + number |
|  | Check ParentCode | ParentCode must exist |
|  | Hide Strain_ID | StrainID hidden in ItemTracker interface |
| Sequencing reaction | Hide Seq_ID | Sequential number hidden in ItemTracker interface |
|  | Set Seq Code | ItemName is ‘Seq’ + number |
|  | Check ParentCode | ParenCode must be present |
|  | Hide Strain_ID | StrainID hidden in ItemTracker interface |
| Oligonucleotide | Hide Oligo_ID | Sequential number hidden in ItemTracker interface |
|  | Set OC Code | ItemName is ‘OC’ + number |
|  | No Spaces | Spaces not allowed in ‘Sequence (5-->3) |
|  | Upper case | upper case only allowed in ‘Sequence (5-->3) |
|  | CertainLettersForSequence | Only letters allowed in ‘Sequence (5-->3) |
| Working stock | Hide WS_ID | Sequential number hidden in ItemTracker interface |
|  | Set WS Code | ItemName is ‘WS’ + number |
