# Supplementary material for: Transforming Microbial Genotyping: A Robotic Pipeline for Genotyping Bacterial Strains
Source: PLoS One. 2012 Oct 29;7(10):e48022. doi: 10.1371/journal.pone.0048022 (PMC3483277; doi:10.1371/journal.pone.0048022)
Supplement: Table S6 — Field rules settings in ItemTracker. (DOCX) [file pone.0048022.s015.docx]

Table S6. Field rules settings in ItemTracker.

| Rule name | Calculation | Result Field | Message Type | Message |
| --- | --- | --- | --- | --- |
| Set B Code | 1 | [ItemName] | Value | Bacteria |
| StrainID error | LEN([STRAINID])==0 |  | Error | You must enter a StrainID |
| Hide Salm_ID | 1 | [Salm_ID] | Setting | Row Hidden#True#100#1 |
| Set S Code | 1 | [ItemName] | Calculation | “S”+LTRIM(STR([Salm_ID])) |
| Check ParentCode | LEN([ParentCode])<1 AND LEN([ItemName])>0 |  | Error | ParentCode must be filled in! |
| Hide Original_ID | 1 | [Original_ID] | Setting | Row Hidden#True#100#1 |
| Hide Altern_ID | 1 | [Altern_ID] | Setting | Row Hidden#True#100#1 |
| Hide Strain_ID | 1 | [StrainID] | Setting | Row Hidden#True#100#1 |
| Hide species | 1 | [species] | Setting | Row Hidden#True#100#1 |
| Hide Lis_ID | 1 | [Lis_ID ] | Setting | Row Hidden#True#100#1 |
| Set L Code | 1 | [ItemName] | Calculation | “S”+LTRIM(STR([Lis_ID])) |
| Hide Ecoli_ID | 1 | [Ecoli _ID] | Setting | Row Hidden#True#100#1 |
| Set E Code | 1 | [ItemName] | Calculation | “E”+LTRIM(STR([Ecoli_ID])) |
| Hide Stock_ID | 1 | [Stock _ID] | Setting | Row Hidden#True#100#1 |
| Set F Code | 1 | [ItemName] | Calculation | “F”+LTRIM(STR([Stock_ID])) |
| Hide DNA_ID | 1 | [DNA _ID] | Setting | Row Hidden#True#100#1 |
| Set D Code | 1 | [ItemName] | Calculation | “D”+LTRIM(STR([DNA_ID])) |
| Hide PCR_ID | 1 | [PCR _ID] | Setting | Row Hidden#True#100#1 |
| Set P Code | 1 | [ItemName] | Calculation | “P”+LTRIM(STR([PCR_ID])) |
| Hide Seq_ID | 1 | [Seq _ID] | Setting | Row Hidden#True#100#1 |
| Set Seq Code | 1 | [ItemName] | Calculation | “Seq”+LTRIM(STR([Seq_ID])) |
| No Spaces | INSTR(1,[Sequence (5-->3)],””) |  | Error | No spaces are allowed! |
| Upper case | UCASE([Sequence (5-->3)]<> [Sequence (5-->3)]) |  | Error | Only upper case allowed! |
| CertainLettersForSequence | Checkval([Sequence (5-->3)]) |  | Error | Some of the characters are not allowed! |
| Hide Oligo_ID | 1 | [Oligo _ID] | Setting | Row Hidden#True#100#1 |
| Set OC Code | 1 | [ItemName] | Calculation | “OC”+LTRIM(STR([Oligo_ID])) |
| Hide WS_ID | 1 | [WS _ID] | Setting | Row Hidden#True#100#1 |
| Set WS Code | 1 | [ItemName] | Calculation | “WS”+LTRIM(STR([WorkingStock_ID])) |
