# Supplementary material for: Transforming Microbial Genotyping: A Robotic Pipeline for Genotyping Bacterial Strains
Source: PLoS One. 2012 Oct 29;7(10):e48022. doi: 10.1371/journal.pone.0048022 (PMC3483277; doi:10.1371/journal.pone.0048022)
Supplement: Table S7 — General Overlord files and visual basic scripts used by LHS1 procedures. (DOCX) [file pone.0048022.s016.docx]

**Table S7. General Overlord files and visual basic scripts used by LHS1 procedures.**

| Identifier | Name of file | Description |
| --- | --- | --- |
| LHS1-1 | Initialise Hardware.ovp | Initializes decapper, dispenser and robot arm |
| LHS1-2 | Move Dispenser Station.ovp | Moves dispenser to position [DispenserStation] = 6 |
| LHS1-3 | Stack1Get.ovp | Picks up tube rack from stack 1 shelf = [current plate] |
| LHS1-4 | Stack1Put.ovp | Puts tube rack onto stack 1 shelf = [current plate] |
| LHS1-5 | Stack2Get.ovp | Picks up tube rack from stack 2 shelf = [current plate] |
| LHS1-6 | Stack2Put.ovp | Puts tube rack onto stack 2 shelf = [current plate] |
| LHS1-7 | DispenserGet.ovp | Gets tube rack from [DispenserPosition] = C2 |
| LHS1-8 | DispenserPut.ovp | Puts tube rack in [DispenserPosition] = C2 |
| LHS1-9 | Remove Caps.ovp | Decaps tube rack |
| LHS1-10 | Replace Caps.ovp | Recaps tube rack |
| LHS1-11 | Select Dispenser Postion.vb | Sets [DispenserStation] equal to [DispenserPosition] |

[CurrentPlate] is a variable that designates the tube rack that is currently being moved by the robot arm for capping/decapping, reading the barcode or pipetting.
