# Supplementary material for: Transforming Microbial Genotyping: A Robotic Pipeline for Genotyping Bacterial Strains
Source: PLoS One. 2012 Oct 29;7(10):e48022. doi: 10.1371/journal.pone.0048022 (PMC3483277; doi:10.1371/journal.pone.0048022)
Supplement: Table S8 — Parameters for filling tube racks. (DOCX) [file pone.0048022.s017.docx]

**Table S8. Parameters for filling tube racks.**

| Dispense volume (µl) | Maximum number of | | Volume of sterile liquid media per reservoir (ml) |
| --- | --- | --- | --- |
|  | tube racks | reservoirs |  |
| 250 | 9 | 3 | 90 |
| 500 | 9 | 3 | 170 |
| 550 | 9 | 3 | 170 |
| 700 | 8 | 4 | 170 |
| 750 | 8 | 4 | 170 |
| 860 | 4 | 4 | 110 |
| 1000 | 4 | 4 | 110 |
