# Supplementary material for: Transforming Microbial Genotyping: A Robotic Pipeline for Genotyping Bacterial Strains
Source: PLoS One. 2012 Oct 29;7(10):e48022. doi: 10.1371/journal.pone.0048022 (PMC3483277; doi:10.1371/journal.pone.0048022)
Supplement: Table S9 — Overlord and Apricot files used by LHS1 for dispensing sterile media. (DOCX) [file pone.0048022.s018.docx]

**Table S9. Overlord and Apricot files used by LHS1 for dispensing sterile media.**

| Identifier | Name of file |
| --- | --- |
| LHS1-12 | !Dispense Sterile Media.ovp |
| LHS1-13 | !Step1-1ml.ovp |
| LHS1-14 | !Step1-250ul.ovp |
| LHS1-15 | !Step1-500ul.ovp |
| LHS1-16 | !Step1-550ul.ovp |
| LHS1-17 | !Step1-700ml.ovp |
| LHS1-18 | !Step1-750ml.ovp |
| LHS1-19 | !Step1-860ul.ovp |
| LHS1-20 | Run Step1_1ml.ovp |
| LHS1-21 | Run Step1_250ul.ovp |
| LHS1-22 | Run Step1_500ul.ovp |
| LHS1-23 | Run Step1_550ul.ovp |
| LHS1-24 | Run Step1_700ul.ovp |
| LHS1-25 | Run Step1_750ul.ovp |
| LHS1-26 | Run Step1_860ul.ovp |
| LHS1-27 | Setup Step1_1ml.ovp |
| LHS1-28 | Setup Step1_500ul.ovp |
| LHS1-29 | Setup Step1_550ul.ovp |
| LHS1-30 | Setup Step1_750ml.ovp |
| LHS1-31 | aspirate 1ml reservoir 1.mp6 |
| LHS1-32 | aspirate 1ml reservoir 3.mp6 |
| LHS1-33 | aspirate 1ml reservoir 4.mp6 |
| LHS1-34 | aspirate 1ml reservoir 5.mp6 |
| LHS1-35 | Aspirate 250ul Reservoir 1.mp6 |
| LHS1-36 | Aspirate 250ul Reservoir 3.mp6 |
| LHS1-37 | Aspirate 250ul Reservoir 4.mp6 |
| LHS1-38 | Aspirate 500ul Reservoir 1.mp6 |
| LHS1-39 | Aspirate 500ul Reservoir 3.mp6 |
| LHS1-40 | Aspirate 500ul Reservoir 4.mp6 |
| LHS1-41 | Aspirate 550ul Reservoir 1.mp6 |
| LHS1-42 | Aspirate 550ul Reservoir 3.mp6 |
| LHS1-43 | Aspirate 550ul Reservoir 4.mp6 |
| LHS1-44 | Aspirate 700ul Reservoir1.mp6 |
| LHS1-45 | Aspirate 700ul Reservoir3.mp6 |
| LHS1-46 | Aspirate 700ul Reservoir4.mp6 |
| LHS1-47 | Aspirate 700ul Reservoir5.mp6 |
| LHS1-48 | Aspirate 750ul Reservoir 1.mp6 |
| LHS1-49 | Aspirate 750ul Reservoir 3.mp6 |
| LHS1-50 | Aspirate 750ul Reservoir 4.mp6 |
| LHS1-51 | Aspirate 750ul Reservoir 5.mp6 |
| LHS1-52 | aspirate 860ul reservoir 1.mp6 |
| LHS1-53 | aspirate 860ul reservoir 3.mp6 |
| LHS1-54 | aspirate 860ul reservoir 4.mp6 |
| LHS1-55 | aspirate 860ul reservoir 5.mp6 |
