# Supplementary material for: Transforming Microbial Genotyping: A Robotic Pipeline for Genotyping Bacterial Strains
Source: PLoS One. 2012 Oct 29;7(10):e48022. doi: 10.1371/journal.pone.0048022 (PMC3483277; doi:10.1371/journal.pone.0048022)
Supplement: Table S10 — Overlord files used by LHS1 for sub-culturing bacterial cultures. (DOCX) [file pone.0048022.s019.docx]

**Table S10. Overlord files used by LHS1 for sub-culturing bacterial cultures.**

| Identifier | Name of file |
| --- | --- |
| LHS1-56 | DispenseSubCultureAndDWP.ovp |
| LHS1-57 | MakeSubCulture.ovp |
| LHS1-58 | MoveDWPToDispenserPos4.ovp |
| LHS1-59 | Scan2DAnd1DBar codes.ovp |
| LHS1-60 | Scan Bar code.ovp |
| LHS1-61 | Scan DNA rack Bar codes.ovp |
| LHS1-62 | Scan second DNA rack Bar codes.ovp |
| LHS1-63 | Scan Tube Rack Bar codes.ovp |
| LHS1-64 | ScanAppropiateBar codes.ovp |
| LHS1-65 | ScanBar codesOnlyTubeRack.ovp |
| LHS1-66 | ScanBar codesTubeRackAndDWP.ovp |
| LHS1-67 | ScanBar codesTubeRackAndTwoDWP.ovp |
| LHS1-68 | Source500Dest700.ovp |
| LHS1-69 | Source500Dest700DWP425.ovp |
| LHS1-70 | Source500Dest700TwiceDWP425.ovp |
| LHS1-71 | Source550Dest500.ovp |
| LHS1-72 | Source550Dest500DWP700.ovp |
| LHS1-73 | Source550Dest700.ovp |
| LHS1-74 | Source750Dest500.ovp |
| LHS1-75 | Source750Dest500DWP700.ovp |
| LHS1-76 | Source750Dest700.ovp |
| LHS1-77 | Source750Dest700DWP425.ovp |
| LHS1-78 | DispenseSubculture.ovp |
