# Supplementary material for: Transforming Microbial Genotyping: A Robotic Pipeline for Genotyping Bacterial Strains
Source: PLoS One. 2012 Oct 29;7(10):e48022. doi: 10.1371/journal.pone.0048022 (PMC3483277; doi:10.1371/journal.pone.0048022)
Supplement: Table S11 — Visual basic scripts used by Overlord for sub-culturing bacteria. (DOCX) [file pone.0048022.s020.docx]

**Table S11. Visual basic scripts used by Overlord for sub-culturing bacteria.**

| Identifier | Name of file | Description |
| --- | --- | --- |
| LHS1-79 | Check 2D Bar code Scan.vb | Checks if all 96 2-D bar-codes were successfully scanned |
| LHS1-80 | CreateDestinationPlateOutputFile.vb | Creates output file with 2-D bar-codes for child rack |
| LHS1-81 | CreateOutPutFileDNARack.vb | Creates output file with 2-D bar-codes for DNA rack |
| LHS1-82 | CreateOutPutFilesecondDNARack.vb | Creates output file with 2-D bar-codes for second DNA rack |
| LHS1-83 | CreateSourcePlateOutputFile.vb | Creates output file with 2-D bar-codes for parent rack |
| LHS1-84 | Delete Old xtr data.vb | Deletes any old files with scanned bar-codes |
| LHS1-85 | ScanNotSuccessful.vb | Writes to file “not successful” |
| LHS1-86 | ScanSuccessful.vb | Writes to file “successful” |
| LHS1-87 | WhichProgramChosen.vb | Obtain user input about choosen procedure and volumes |
