# Supplementary material for: Transforming Microbial Genotyping: A Robotic Pipeline for Genotyping Bacterial Strains
Source: PLoS One. 2012 Oct 29;7(10):e48022. doi: 10.1371/journal.pone.0048022 (PMC3483277; doi:10.1371/journal.pone.0048022)
Supplement: Table S12 — Protocols used for configuring the dispenser of LHS1 for various labware and volumes for the sub-culturing procedure. (DOCX) [file pone.0048022.s021.docx]

**Table S12. Protocols used for configuring the dispenser of LHS1 for various labware and volumes for the sub-culturing procedure.**

| Identifier | Name of files | Volume  parent rack (µl) | Volume  child rack (µl) | Volume transferred into deep well plate (µl) | |
| --- | --- | --- | --- | --- | --- |
|  |  |  |  | Sterile media | Bacterial culture |
| LHS1-88 | !Source750Dest700.mp6 | 750 | 700 | - | - |
| LHS1-89 | !Source750Dest500.mp6 | 750 | 500 | - | - |
| LHS1-90 | !Source550Dest700.mp6 | 550 | 700 | - | - |
| LHS1-91 | !Source550Dest500.mp6 | 550 | 500 | - | - |
| LHS1-92 | !Source500Dest700.mp6 | 500 | 700 | - | - |
| LHS1-93 | !DWP700Source750Dest500.mp6 | 750 | 500 | 700 | 50 |
| LHS1-94 | !DWP700Source550Dest500.mp6 | 550 | 500 | 700 | 50 |
| LHS1-95 | !DWP425Source750Dest700.mp6 | 750 | 700 | 425 | 25 |
| LHS1-96 | !DWP425Source500Dest700.mp6 | 500 | 700 | 425 | 25 |
| LHS1-97 | !TwiceDWP425Source500Dest700.mp6 | 500 | 700 | 2 x 425 | 2 x 25 |
