# Supplementary material for: Transforming Microbial Genotyping: A Robotic Pipeline for Genotyping Bacterial Strains
Source: PLoS One. 2012 Oct 29;7(10):e48022. doi: 10.1371/journal.pone.0048022 (PMC3483277; doi:10.1371/journal.pone.0048022)
Supplement: File S1 — Configuration of ItemTracker. (DOCX) [file pone.0048022.s022.docx]

**Supplementary File S1**

Configuration of ItemTracker

1. Introduction
2. ItemTypes and properties
3. Field rules
4. SQL table structure and views

# Introduction

This document describes how we integrated the commercial software ItemTracker (ItemTracker Ltd., South Nutfield, UK) in a microbiology laboratory. After installation of ItemTracker ‘ItemTypes’ can be defined to which multiple ItemProperties can be assigned. Some common properties are pre-defined and need not to be changed (Table S3). Two additional common properties, ‘Selected’ and ‘Selected by’, were defined that also apply to all items (Table S4).

# ItemTypes and properties

Ten novel ItemTypes were defined (Table S4). ItemTypes that are children of other ItemTypes inherit all their ItemProperties. All ItemProperties must have a unique designation and can only be assigned to one ItemType. ItemTypes “Salmonella”, “Listeria” and “Ecoli” were defined as children of ‘Bacteria’. All other ItemTypes were created without parental links to any other ItemType.

# Field rules

Rules were applied to selected ItemTypes, e.g. when only certain characters are acceptable for an ItemProperty such as for the DNA sequence of an oligonucleotide or to generate a code letter followed by a sequential number for the unique ItemName of each item (Table S5, Table S6). All items that are derived from ‘Bacteria’ inherit its StrainID, which was implemented by running the following SQL trigger rule on the database:

-----------------------------------------------------------------------------

USE [ItemTracker]

GO

if exists (select * from dbo.sysobjects where id = object_id(N'[dbo].[UpdateParentCode]') and OBJECTPROPERTY(id, N'IsTrigger') = 1)

drop trigger [dbo].[UpdateParentCode]

GO

CREATE TRIGGER UpdateParentCode ON dbo.Items

FOR INSERT, UPDATE

AS

DECLARE @FieldID INT, @TypeID int

SELECT @FieldID = TypeID FROM ItemProperty WHERE Name = 'Strain_ID'

IF UPDATE(ParentCode)

IF EXISTS(SELECT 1 FROM ItemValue IV, Inserted Ins WHERE IV.ItemID = Ins.ItemID AND IV.SeqNo = Ins.SeqNo AND IV.FieldID = @FieldID) BEGIN

UPDATE IV1 SET IV1.FieldValue = IV2.FieldValue FROM ItemValue IV1, Inserted Ins, Items I, ItemValue IV2

WHERE IV1.ItemID = Ins.ItemID AND IV1.SeqNo = Ins.SeqNo AND Ins.ParentCode = I.ItemID

AND ISNUMERIC(Ins.ParentCode) = 1 AND I.ItemID = IV2.ItemID AND I.SeqNo = IV2.SeqNo

AND IV1.FieldID = @FieldID AND IV2.FieldID = @FieldID

END ELSE BEGIN

INSERT INTO ItemValue(ItemID, SeqNo, FieldID, FieldValue)

SELECT Ins.ItemID, Ins.SeqNo, @FieldID, IV.FieldValue FROM ItemValue IV, Items I, Inserted Ins

WHERE IV.ItemID = I.ItemID AND IV.SeqNo = I.SeqNo AND I.ItemID = Ins.ParentCode

AND ISNUMERIC(Ins.ParentCode) = 1 AND IV.FieldID = @FieldID

END

-----------------------------------------------------------------------------

# SQL table structure and views

Scripts written in Python were commonly used to update, insert or retrieve data in ItemTracker. The commercial version of ItemTracker contains the tables shown in Fig. S4, which were manipulated by these scripts. In order to facilitate easier retrieval of data about items, their properties and locations, we created two SQL views that combine data from various tables:

View: LocationView

-----------------------------------------------------------------------------

create view LocationView as

select V.ItemID,

VI.Level2Name as Freezer,

IV.Level3Name as Rack,

III.Level4Name as Shelf,

II.Level5Name as PlateRack,

I.Pos as Position

from Locations I,Level5 II, Level4 III, Level3 IV,Items V, Level2 VI

where I.Level5ID = II.Level5ID

and II.Level4ID = III.Level4ID

and III.Level3ID = IV.Level3ID

and IV.Level2ID = VI.Level2ID

and V.LocationID = I.LocationID

-----------------------------------------------------------------------------

View: ItemTable

-----------------------------------------------------------------------------

create view ItemTable as

select I.ItemID, I.ItemName,I.CreatorID,I.LocationID,I.ExtBarcode,I.InputDate, I.ParentCode, I.SeqNo, III.Username as UserName,IV.Name as ItemType,

ItemTable.[1] as DNAID,

ItemTable.[4] as StockID,

ItemTable.[7] as StrainID,

ItemTable.[8] as AlternID,

ItemTable.[9] as LisID,

ItemTable.[10] as SalmID,

ItemTable.[11] as PCRID,

ItemTable.[12] as OligoID,

ItemTable.[13] as DNAconcentrationngul,

ItemTable.[14] as PrimersP,

ItemTable.[16] as GeneFragmentPCR,

ItemTable.[17] as TypeofCultureL,

ItemTable.[18] as viabilityL,

ItemTable.[19] as TypeofCultureS,

ItemTable.[21] as OriginalID,

ItemTable.[23] as EcoliID,

ItemTable.[24] as FreezingMedium,

ItemTable.[25] as TypeofCultureE,

ItemTable.[26] as Species,

ItemTable.[27] as AdjustedConcn,

ItemTable.[28] as Date,

ItemTable.[29] as VolumeDNA,

ItemTable.[30] as viabilityS,

ItemTable.[31] as viabilityE,

ItemTable.[32] as viabilityF,

ItemTable.[33] as Oligoname,

ItemTable.[34] as Organism,

ItemTable.[35] as Sequence,

ItemTable.[36] as GeneFragmentSNP,

ItemTable.[37] as Purpose,

ItemTable.[38] as MeltingTemperature,

ItemTable.[39] as Supplier,

ItemTable.[40] as DateofOrder,

ItemTable.[41] as Direction,

ItemTable.[42] as Project,

ItemTable.[43] as AntitagRegion,

ItemTable.[44] as ListeriaStatus,

ItemTable.[45] as SalmonellaStatus,

ItemTable.[46] as Ecolistatus,

ItemTable.[52] as FrozenStatus,

ItemTable.[53] as DNAStatus,

ItemTable.[54] as PCRStatus,

ItemTable.[55] as ProductLength,

ItemTable.[56] as PartnerOligo,

ItemTable.[57] as Selected,

ItemTable.[58] as SelectedBy,

ItemTable.[59] as VolumeFrozenStock,

ItemTable.[60] as AnnealingTemperatureP,

ItemTable.[61] as NumberOfCyclesP,

ItemTable.[62] as WellFormatP,

ItemTable.[65] as VolumeP,

ItemTable.[71] as SequencingPrimer,

ItemTable.[72] as StatusSeq,

ItemTable.[74] as SpeciesWS,

ItemTable.[75] as GeneWS,

ItemTable.[76] as PrimersWS,

ItemTable.[77] as VolumeWS,

ItemTable.[78] as ConcentrationWS,

ItemTable.[79] as BionumericsKey,

ItemTable.[80] as GeneSeqReaction,

ItemTable.[81] as VolumeSeqReaction,

ItemTable.[82] as DirectionSeq

from Items I,Users III,ItemType IV,ItemValue II

PIVOT

(max(II.FieldValue)for II.FieldID in ([1],[4],[7],[8],[9],[10],[11],[12],[13],[14],[16],[17],[18],[19],[21],[23],[24],[25],[26],[27],[28],[29],[30],[31],[32],[33],[34],[35],[36],[37],[38],[39],[40],[41],[42],[43],[44],[45],[46],[48],[49],[50],[51],[52],[53],[54],[55],[56],[57],[58],[59],[60],[61],[62],[65],[67],[68],[71],[72],[74],[75],[76],[77],[78],[79],[80],[81],[82]))

as ItemTable

where I.ItemID = ItemTable.ItemID and I.SeqNo = ItemTable.SeqNo and III.UserID = I.CreatorID and I.ItemTypeID = IV.TypeID

-----------------------------------------------------------------------------
