# Supplementary material for: Transforming Microbial Genotyping: A Robotic Pipeline for Genotyping Bacterial Strains
Source: PLoS One. 2012 Oct 29;7(10):e48022. doi: 10.1371/journal.pone.0048022 (PMC3483277; doi:10.1371/journal.pone.0048022)
Supplement: File S2 — Setup of liquid handling system 1 (LHS1). (DOCX) [file pone.0048022.s023.docx]

**Supplementary File S2**

Setup of liquid handling system 1 (LHS1)

1. Introduction
2. Dispense sterile media
3. Subculture bacteria

# Introduction

Liquid handler system 1 (LHS1) is a composite system of three robotic devices: a capper/decapper, a robot arm and a dispenser. The robot arm and the capper/decapper are controlled through Overlord, a commercial software (Process Analyses and Automation (PAA), Farnborough, UK) which can execute a variety of protocols that can be pre-set in the dispenser (Apricot, FluidX, Nether Alderley, UK). Overlord can also pause or continue execution of the dispenser. Pauses in pipetting protocols are important because the composite system only includes one capper/decapper. Thus, transferring liquid from one tube rack to a second demands a pause in the pipetting procedure to allow intermediate capping of the first rack und decapping of the second as well as moving them from one position to another. The three components are installed within a custom built class 2 safety cabinet which also includes 1-D and 2-D bar-code readers, two stack units which each allows storing of 5 tube racks (designated 1-10) and a rest position (Fig. S5). The dispenser has a movable stage with 5 positions ([DispenserStation]=1-5) as well as an initializing position designated 6.

The protocols allow the user to specify certain variations such as different volumes or numbers of racks to be pipetted. A choice between these options is provided by a central procedure within Overlord (Fig. S6) or a Python script (Fig. S7), which guide the user through the different options. This renders using the robot easier for personnel and also allows transparent addition of new protocols.

# Dispense sterile media

This procedure can fill up to nine tube racks with one of seven different volumes of sterile liquid media (Table S8). Liquid media is provided in disposable plastic reservoirs (57-1202S, FluidX, UK). The volumes to be dispensed and the number of tube racks are encoded in multiple Overlord files (.ovp) which interact with distinct dispenser protocols (.mp6) that specify the reservoir positions for aspiration and the pipetting parameters (Table S9). A schematic overview of the relationship between these files is shown in Fig. S8. Sterile empty tube racks are placed in the stack units in sequential order, first in stack unit A (position 1 through 5), and similarly in stack unit B. Sterile reservoirs are placed in dispenser positions C1 and C3-C5 and are filled manually with sterile liquid media (Table S8). Position C2 is reserved as a working position for a decapped tube rack.

# Subculture bacteria

Script B1 with a graphical user interface (GUI) is used to guide users through the process of sub-culturing bacterial cultures from frozen stocks in a tube rack into a new tube rack previously filled with sterile media and optionally to also inoculate one or two deep well plates which are filled with sterile media during this procedure (Fig. S7). Script B1 run a variety of Overlord procedures (Table S7, Table S10-11) and Python Script B2 or B3 depending on user parameters. The dispenser is configured with protocols in Table S12. A schematic overview of the relationship between these files is shown in Fig. S9.
